# Oxidative stress induced by Cu nutritional disorders in *Citrus* depends on nitrogen and calcium availability

**DOI:** 10.1038/s41598-018-19735-x

**Published:** 2018-01-26

**Authors:** Franz Walter Rieger Hippler, Rodrigo Marcelli Boaretto, Veronica Lorena Dovis, José Antônio Quaggio, Ricardo Antunes Azevedo, Dirceu Mattos-Jr

**Affiliations:** 1 Centro de Citricultura Sylvio Moreira, Instituto Agronômico, Rod. Anhanguera, km 158, CP 04, CEP 13490-970 Cordeirópolis, SP Brazil; 2 Centro de Solos e Recursos Ambientais, Instituto Agronômico, Av. Barão de Itapura, 1481, CP 28, CEP 13020-902 Campinas, SP Brazil; 3 Departamento de Genética, Escola Superior de Agricultura Luiz de Queiroz, CP 9, Universidade de São Paulo, 13418-900 Piracicaba, SP Brazil

## Abstract

Nutritional stress caused by copper (Cu) deficiency or toxicity affects fruit production of citrus orchards worldwide, but this could be minimised by fine-tuned fertilisation in the orchards. Two experiments were performed aiming to evaluate the photosynthetic capacity and the antioxidant enzyme activities of Swingle citrumelo seedlings, grown in nutrient solution (NS) with two levels of nitrogen (N) in the first experiment (adequate-N and high-N) and two levels of calcium (Ca) in the second (low-Ca and adequate-Ca). Plants were then exposed to various Cu levels (low, medium and high) for 15 days. Plants under Cu-toxicity exhibited specific effects on reactive oxygen species formation and root-to-shoot plant signalling. Copper absorption was greater with increased Cu concentration in the NS, which reduced plant biomass accumulation, gas exchange measurements, the activity of nitrate reductase and affected Cu partitioning between roots and shoots. Despite these effects, oxidative stress induced by excess-Cu was reduced at the highest N dose when compared to control and, on the contrary, increased with low-Ca supply. Therefore, a rational supply of N or Ca minimises Cu-induced stress damages to roots and leaves of plants, by directly enhancing the antioxidant system and protecting the associated antioxidative enzyme activities, whilst maintaining photosynthesis.

## Introduction

Copper (Cu) deficiency impairs the growth of young non-bearing citrus trees and this is mainly associated with a limited supply of the metal by Cu-based pesticides, as these products are barely used in the first years of grove establishment in the field. Conversely, the intensive use of such pesticides to control citrus diseases in bearing trees, causes accumulation of Cu in soils, limiting citrus production^1,2^. Despite detrimental effects of excess Cu on soil quality and tree health in citrus groves, the inputs are likely to increase because of the widespread occurrence of citrus canker (*Xanthomonas citri* subsp. *citri*^3^) and citrus black spot (*Phyllosticta citricarpa*^4^) in major citrus production areas in Brazil. In the field, Cu-based pesticides can reach an amount of 30 kg ha^−1^ year^−1^ of the metal^1,3^, in which most of the Cu sprayed to the leaves is deposited on the soil surface.

Accordingly, a comprehensive understanding of the responses of citrus trees to Cu toxicity and a definition of better management practices, aiming to reduce deleterious effects of this metal on plants, is critical for the sustainability of the citrus industry. Therefore, we argue that a sound approach to improve plant tolerance to high Cu levels in the root medium would be achieved by the proper nutritional management of trees in problem soils. For instance, phosphorous (P) deficiency predisposes *Citrus* trees to Cu toxicity and an adequate supply of this nutrient ameliorates the detrimental effects of the metal on root functioning^5^. Furthermore, other studies have demonstrated beneficial effects of a sufficient nutrient supply on plant tolerance to a number of mineral stresses^6,7^.

Nitrogen (N) and calcium (Ca) availability is inherently low in tropical soils, whereas, such nutrients are absorbed in greatest amounts by citrus trees^8^, and thus, play a key role in fruit yield^9^. However, poor plant growth under Cu deficiency might be more pronounced under high N fertilisation^10^. Excess Cu increases reactive oxygen species (ROS) production and compromises absorption and assimilation of nitrate due to the inhibition of selected enzymatic processes caused by Cu binding to the cysteine residue in the active site of enzymes involved in regulating N assimilation^11^. Nitrogen is a key element for plant growth^12,13^ and higher levels of N supply can increase protein contents and activities of enzymes, such as superoxide dismutase (SOD), catalase (CAT) and guaiacol peroxidase (POX)^14^, which play a role in alleviating the deleterious effects of metal-induced stress conditions in plants. Excess Cu also diminishes Ca absorption in roots by competing for absorption sites and reducing the H^+^-ATPase activity^15,16^. Additionally, an adequate Ca supply improves the integrity of cell membranes and cell walls^17^, minimising deleterious effects of lower cell lignification under Cu deficiency^18^ or reducing uptake of this metal by roots, as well as damages caused by ROS^2,19^.

Accordingly, we hypothesise that a rational application of N or Ca alleviates biochemical and physiological damages caused by Cu-stress in plants. In this context, investigating the effect of an adequate or even additional supply of nutrients, such as N or Ca, on the responses of plants to different Cu supply, is a critical step. Therefore, this study aimed to evaluate how the supply of N or Ca affect the photosynthetic and antioxidant activities of citrus plants after exposure to limiting growth Cu concentrations in nutrient solution (NS).

## Results

### Plant growth

**Figure 1:**
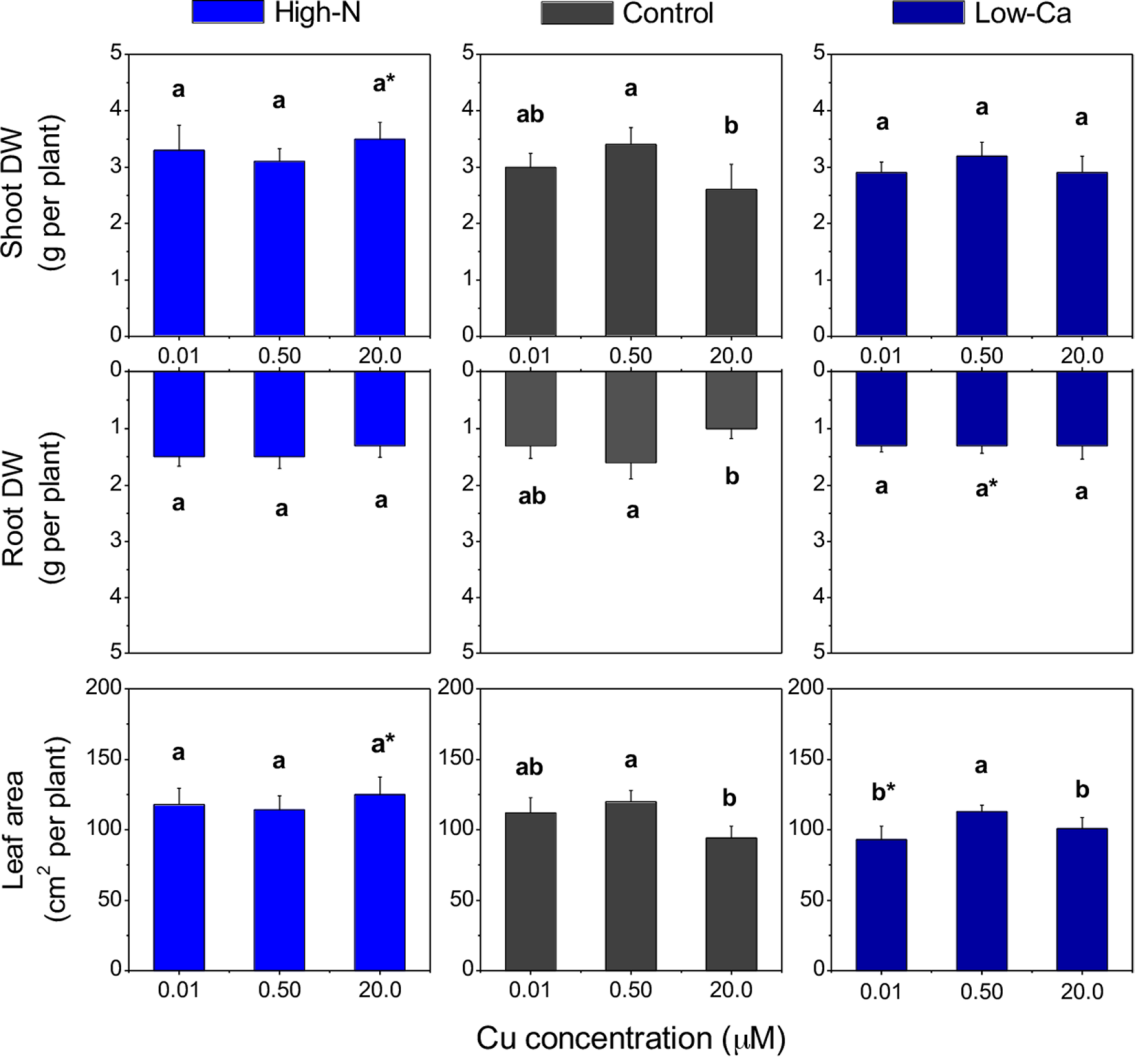
Dry weight (DW) and leaf area of Swingle citrumelo seedlings grown in nutrient solution with different levels of nitrogen (N; Experiment 1) or calcium (Ca; Experiment 2), followed by 15 days in various copper (Cu) concentrations. Legend: high-N: 16.4 mM N; control: 9.4 mM N and 5.7 mM Ca; low-Ca: 1.0 mM Ca; Means of Cu levels followed by different lowercase letters are significantly different by Tukey’s test (*p* < 0.05). Copper levels between High-N and Control or between Low-Ca and Control followed by an asterisk (*) are significantly different by Tukey’s test (*p* < 0.05).

Dry weight of shoots and roots, and the leaf area of control plants decreased with the highest Cu concentration in the NS compared to the medium Cu level (Fig. 1). Whereas no differences were observed for biomass of plants with high-N in the NS, in the Ca-experiment, low-Ca plants exhibited lower leaf area even at the lowest Cu concentration in the NS when compared to control ones (Fig. 1).

### Copper absorption, partitioning and plant nutritional status

**Figure 2:**
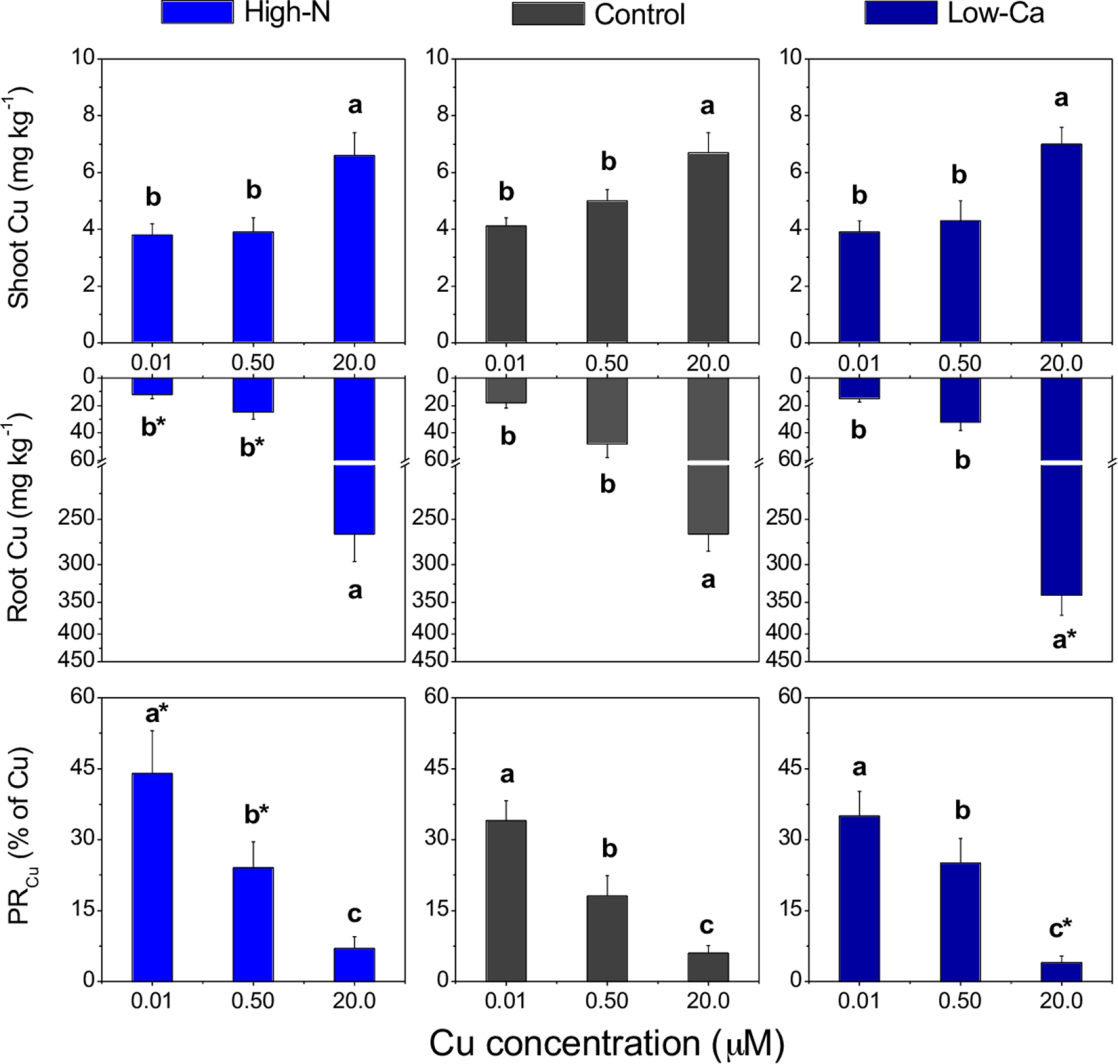
Copper (Cu) concentration and partition ratio of Cu (PR_Cu_) in Swingle citrumelo seedlings grown in nutrient solution with different levels of nitrogen (N; Experiment 1) or calcium (Ca; Experiment 2), followed by 15 days in various Cu concentrations. Legend: high-N: 16.4 mM N; control: 9.4 mM N and 5.7 mM Ca; low-Ca: 1.0 mM Ca; Means of Cu levels followed by different lowercase letters are significantly different by Tukey’s test (*p* < 0.05). Copper levels between High-N and Control or between Low-Ca and Control followed by an asterisk (*) are significantly different by Tukey’s test (*p* < 0.05).

Plant Cu concentration increased 1.8-fold in shoots and 22.3-fold in roots when the highest level of the metal was added in the NS compared to the lowest level (Fig. 2). However, the high-N plants, when in the NS with 0.01 or 0.50 µM Cu, contained lower Cu concentration in the roots than the control plants (Fig. 2). Conversely, low-Ca plants contained the highest Cu concentrations in roots when grown with 20.0 µM Cu (335 mg kg^−1^ Cu; Fig. 2). As a consequence of the high Cu accumulation in roots compared to shoots, the PR_Cu_ decreased, particularly in low-Ca plants with 20.0 µM Cu (Fig. 2). On the contrary, when grown with 0.01 or 0.50 µM Cu in the NS, high-N plants exhibited a higher PR_Cu_ compared to the control (Fig. 2).

Compared to the control plants, high-N plants contained higher N concentration only in roots, while the low-Ca plants contained lower Ca concentrations in roots and shoots (Supplementary File, Fig. S1). Although the Cu concentrations in the NS did not affect either N or Ca total concentrations in plant parts, we observed higher N-NO_3_ concentration in the shoots of high-N plants when grown with NS containing 20.0 than with 0.01 or 0.50 µM Cu (Supplementary File, Fig. S2). Furthermore, high-N plants contained higher levels of N-NO_3_ and N-NH_4_ in the shoots when compared with the control and no difference was observed in the N-NH_4_ concentration as the Cu in the NS was increased (Supplementary File, Fig. S2).

### Activity of NRase

**Figure 3:**
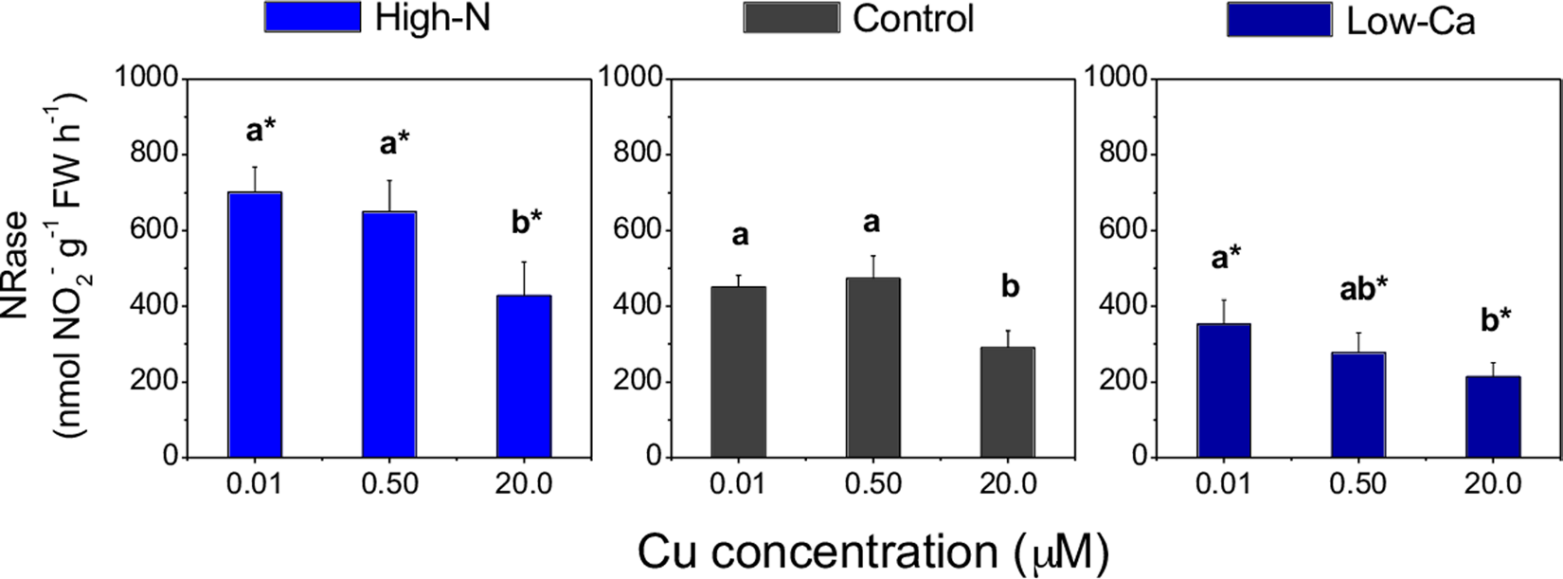
Nitrate reductase (NRase) activity in leaves of Swingle citrumelo seedlings, grown in nutrient solution with different levels of nitrogen (N; Experiment 1) or calcium (Ca; Experiment 2), and followed by15 days in various copper (Cu) concentrations. Legend: high-N: 16.4 mM N; Control: 9.4 mM N and 5.7 mM Ca; low-Ca: 1.0 mM Ca; Means of Cu levels followed by different lowercase letters are significantly different by Tukey’s test (*p* < 0.05). Copper levels between High-N and Control or between Low-Ca and Control followed by an asterisk (*) are significantly different by Tukey’s test (*p* < 0.05).

Copper toxicity decreased the NRase activity in leaves in both experiments (Fig. 3). However, compared to the control plants, plants grown with high-N exhibited a higher NRase activity, while those in the low-Ca condition presented the lowest activity of this enzyme (Fig. 3).

### Leaf gas exchange and photochemistry activity

**Figure 4:**
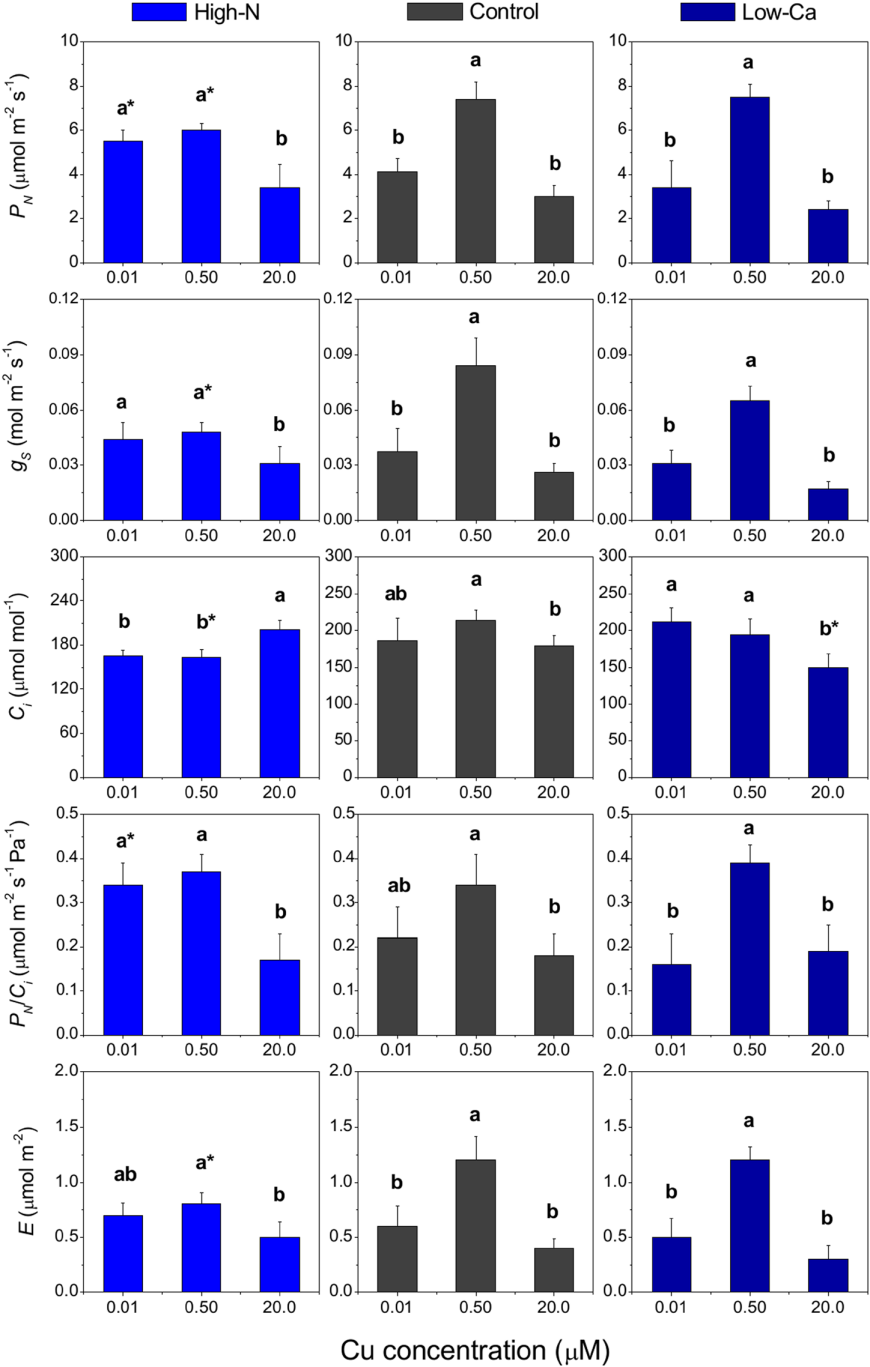
Photosynthetic rate (*P*_*N*_), stomatal conductance (*g*_*S*_), internal CO_2_ concentration (*C*_*i*_), instantaneous carboxylation efficiency (*P*_*N*_/*C*_*i*_) and transpiration (*E*) in leaves of Swingle citrumelo seedlings grown in nutrient solution with different levels of nitrogen (N; Experiment 1) or calcium (Ca; Experiment 2), and followed by 15 days in various copper (Cu) concentrations. Legend: high-N: 16.4 mM N; control: 9.4 mM N and 5.7 mM Ca; low-Ca: 1.0 mM Ca; Means of Cu levels followed by different lowercase letters are significantly different by Tukey’s test (*p* < 0.05). Copper levels between High-N and Control or between Low-Ca and Control followed by an asterisk (*) are significantly different by Tukey’s test (*p* < 0.05).

Diffusive processes of photosynthesis were also affected by Cu concentrations, whereby Cu toxicity reduced the *P*_*N*_, *g*_*S*_, *E* and *P*_*N*_/*C*_*i*_ up to 50% in both experiments, for plants in 0.50 µM Cu (Fig. 4). Furthermore, low-Ca plants under Cu excess also exhibited a reduction of *C*_*i*_ compared to the controls (Fig. 3). In the Ca-experiment, plants that were grown with 0.01 µM Cu exhibited intermediate *P*_*N*_, *g*_*S*_ and *E* values relative to those grown at the higher Cu concentrations (Fig. 4).

**Figure 5:**
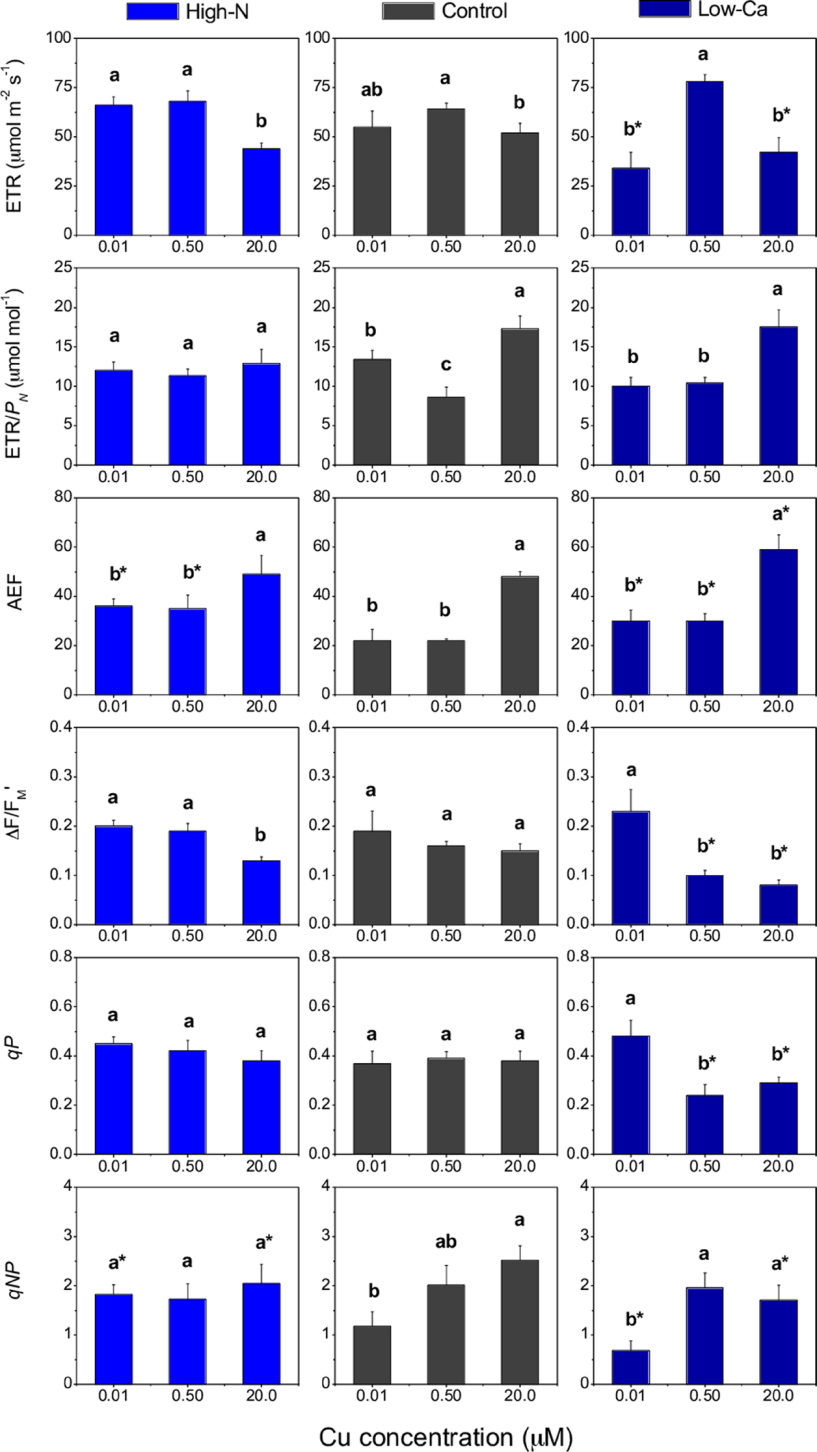
Apparent electron transport rate (ETR), ratio between ETR and CO_2_ assimilation (ETR/*P*_*N*_), alternative electron flow (AEF), the effective quantum yield of photosystem II (∆F/F_M_′), and the photochemical (*qP*) and non-photochemical quenching (*qNP*) in leaves of Swingle citrumelo seedlings grown in nutrient solution with different levels of nitrogen (N; Experiment 1) or calcium (Ca; Experiment 2), and followed by 15 days in various copper (Cu) concentrations. Legend: high-N: 16.4 mM N; control: 9.4 mM N and 5.7 mM Ca; low-Ca: 1.0 mM Ca; Means of Cu levels followed by different lowercase letters are significantly different by Tukey’s test (*p* < 0.05). Copper levels between High-N and Control or between Low-Ca and Control followed by an asterisk (*) are significantly different by Tukey’s test (*p* < 0.05).

**Figure 6:**
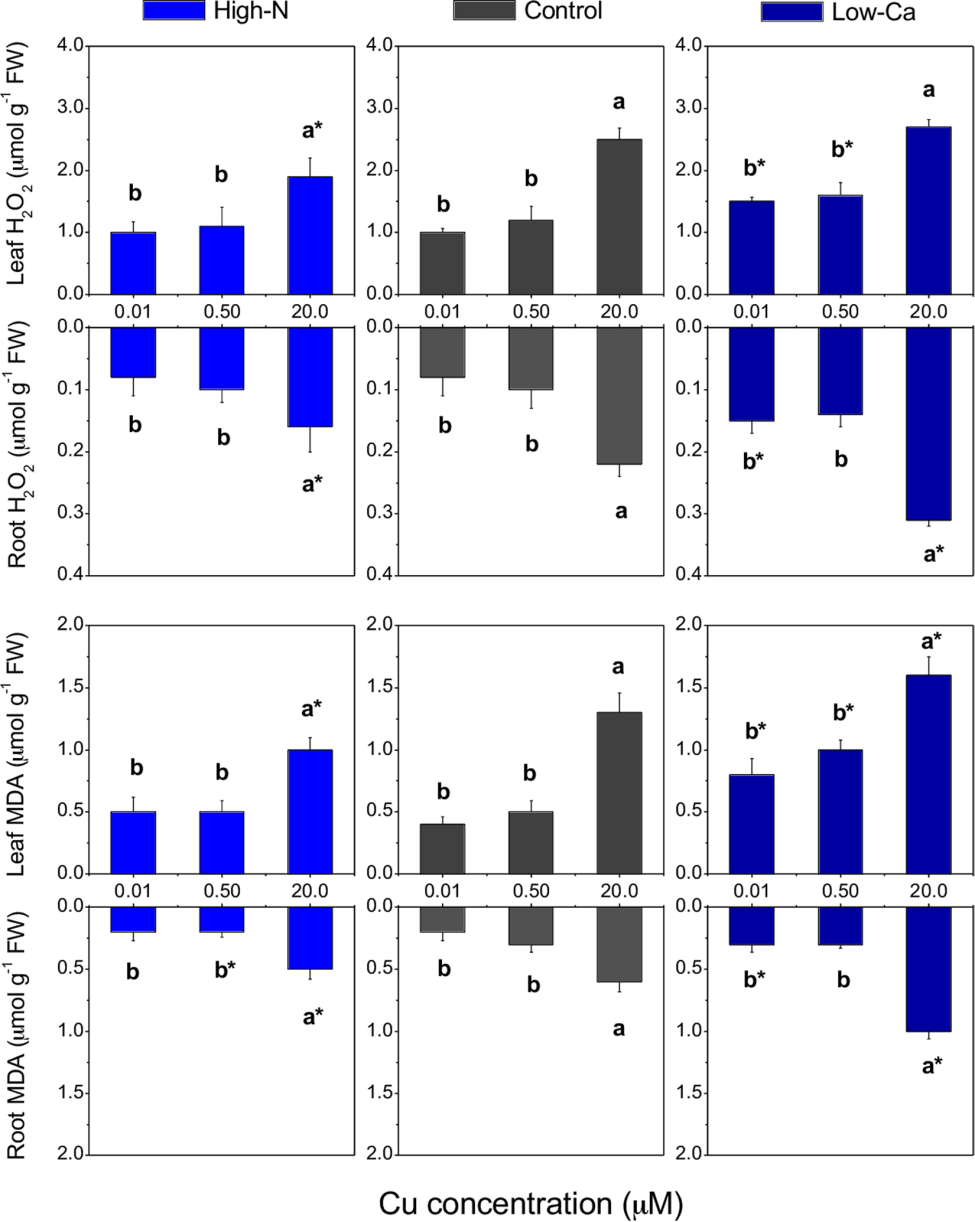
Hydrogen peroxide (H_2_O_2_) concentration and lipid peroxidation (malondialdehyde, MDA) in Swingle citrumelo seedlings grown in nutrient solution with different levels of nitrogen (N; Experiment 1) or calcium (Ca; Experiment 2), and followed by 15 days in various copper (Cu) concentrations. Legend: high-N: 16.4 mM N; control: 9.4 mM N and 5.7 mM Ca; low-Ca: 1.0 mM Ca; Means of Cu levels followed by different lowercase letters are significantly different by Tukey’s test (*p* < 0.05). Copper levels between High-N and Control or between Low-Ca and Control followed by an asterisk (*) are significantly different by Tukey’s test (*p* < 0.05).

The ETR was reduced in plants supplied with the highest Cu level in both experiments compared to plants grown with 0.50 µM Cu (Fig. 5). However, low-Ca plants also exhibited lower ETR values when grown with 0.01 µM Cu compared to the 0.50 µM Cu or to the control plants (Fig. 5). The ETR/*P*_*N*_ ratio increased in the control plants grown in a Cu excess, as well in the lowest Cu concentration in the NS (Fig. 6), whereas the low-Ca plants presented higher ETR/*P*_*N*_ only under Cu toxicity (Fig. 6). The AEF was higher in plants grown with 20.0 than with 0.01 or 0.50 µM Cu in the NS (Fig. 5). However, the control plants exhibited a lower AEF than high-N and low-Ca plants, despite the varying Cu concentration in the NS (Fig. 5).

The ∆F/F_M_′ values decreased under Cu toxicity in the high-N and low-Ca plants, respectively, but not in the controls (Fig. 5). The *qP* decreased only in low-Ca plants with 0.50 or 20.0 µM Cu in the NS, compared with the lowest Cu and the control plants (Fig. 5). Conversely, the *qNP* increased in high-N plants with 0.01 µM Cu relative to the controls but also in the control plants grown in NS with 20.0 µM Cu compared to high-N plants (Fig. 5). In the Ca-experiment, the *qNP* increased in plants with 20.0 µM Cu in both the control and low-Ca plants compared to the lowest Cu level in the NS (Fig. 5).

### H_2_O_2_ and MDA levels

The H_2_O_2_ and MDA contents were higher in leaves and roots of plants grown with 20.0 µM Cu than any of the other Cu concentrations (Fig. 6). However, the H_2_O_2_ concentrations in the leaves and roots, as well as the MDA levels in the leaves, were lower in high-N than in control plants, both under Cu toxicity (Fig. 6). The opposite was verified in low-Ca plants, which exhibited higher concentrations of H_2_O_2_ and MDA in leaves and roots than the control plants when grown in 0.01 or 20.0 µM Cu (Fig. 6).

### Antioxidant enzyme activities

The total protein content in leaves (8.6 ± 0.7 mg g^−1^ FW) and roots (5.3 ± 0.6 mg g^−1^ FW) did not vary with the different levels of Cu, N or Ca supplied (data not shown).

**Figure 7:**
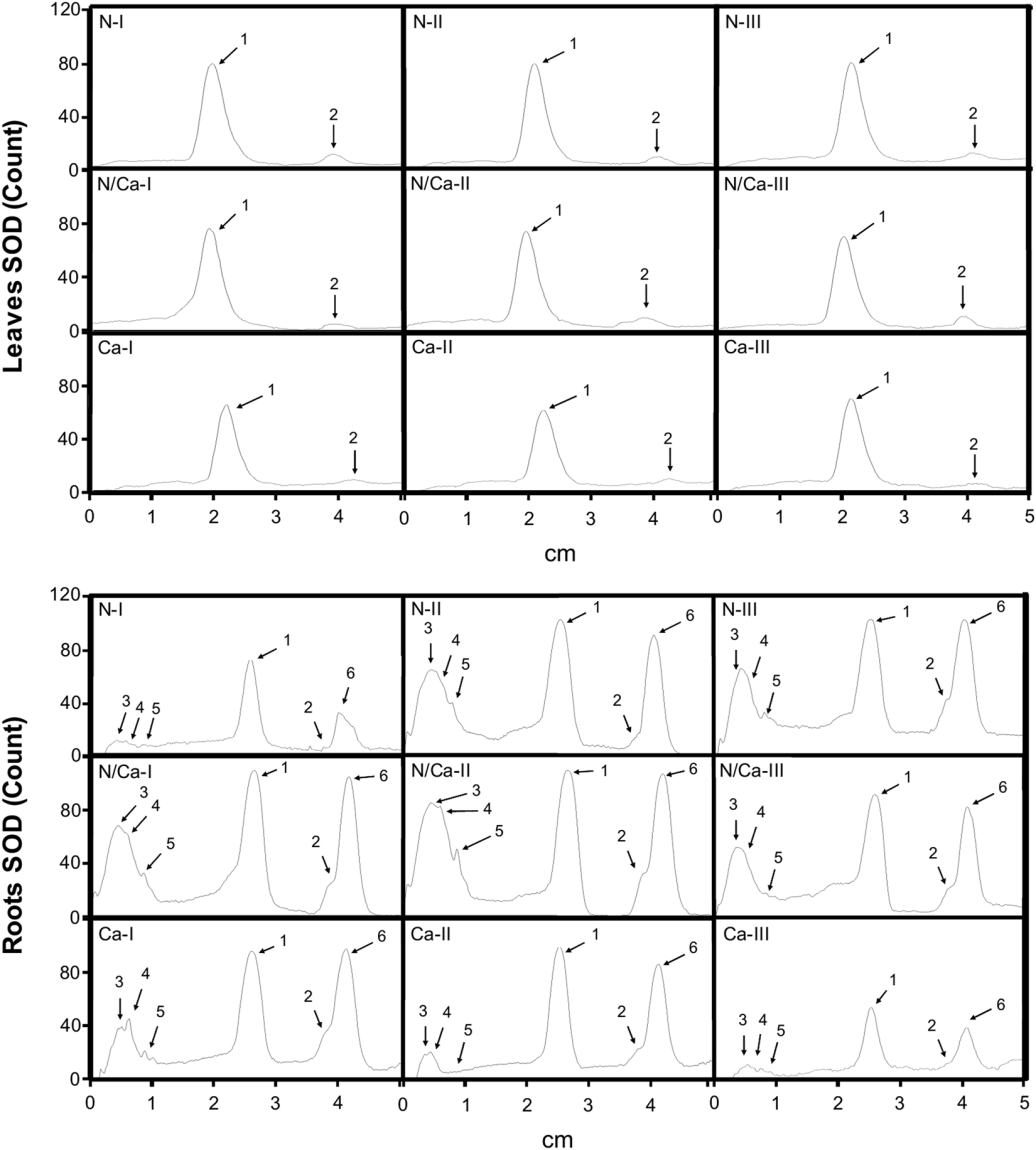
Polyacrylamide gel electrophoresis (PAGE 12%) densitometry of the superoxide dismutase (SOD) activity in leaves and roots of Swingle citrumelo seedlings grown in nutrient solution with different levels of nitrogen (N; Experiment 1) or calcium (Ca; Experiment 2), and followed by 15 days in various copper (Cu) concentrations. Legend: N – high-N; N/Ca – control; Ca – low-Ca; I – 0.01 µM Cu; II – 0.50 µM Cu; III – 20.0 µM Cu; 1 – Mn-SOD; 2 – Cu/Zn-SOD; 3 – Fe-SOD I; 4 – Fe-SOD II; 5 – Fe-SOD III; and 6 – Cu/Zn-SOD II.

In leaves, the activities of the SOD isoforms did not vary with treatments (Fig. 7; Supplementary Info, Fig. S3), except for low-Ca plants that exhibited a decrease in the activities of Mn-SOD isoforms compared to the control (Fig. 7). In roots, the highest Cu level in the NS decreased the activities of the Fe-SOD II and III isoforms (Fig. 7). Furthermore, the activities of the SOD isoforms were lower in high-N plants grown with 0.01 µM Cu compared to the control, whereas low-Ca plants exhibited a lower activity of the Fe-SOD isoforms II and III when grown with 0.50 µM Cu, and a lower total SOD activity with 20.0 µM Cu (Fig. 7).

**Figure 8:**
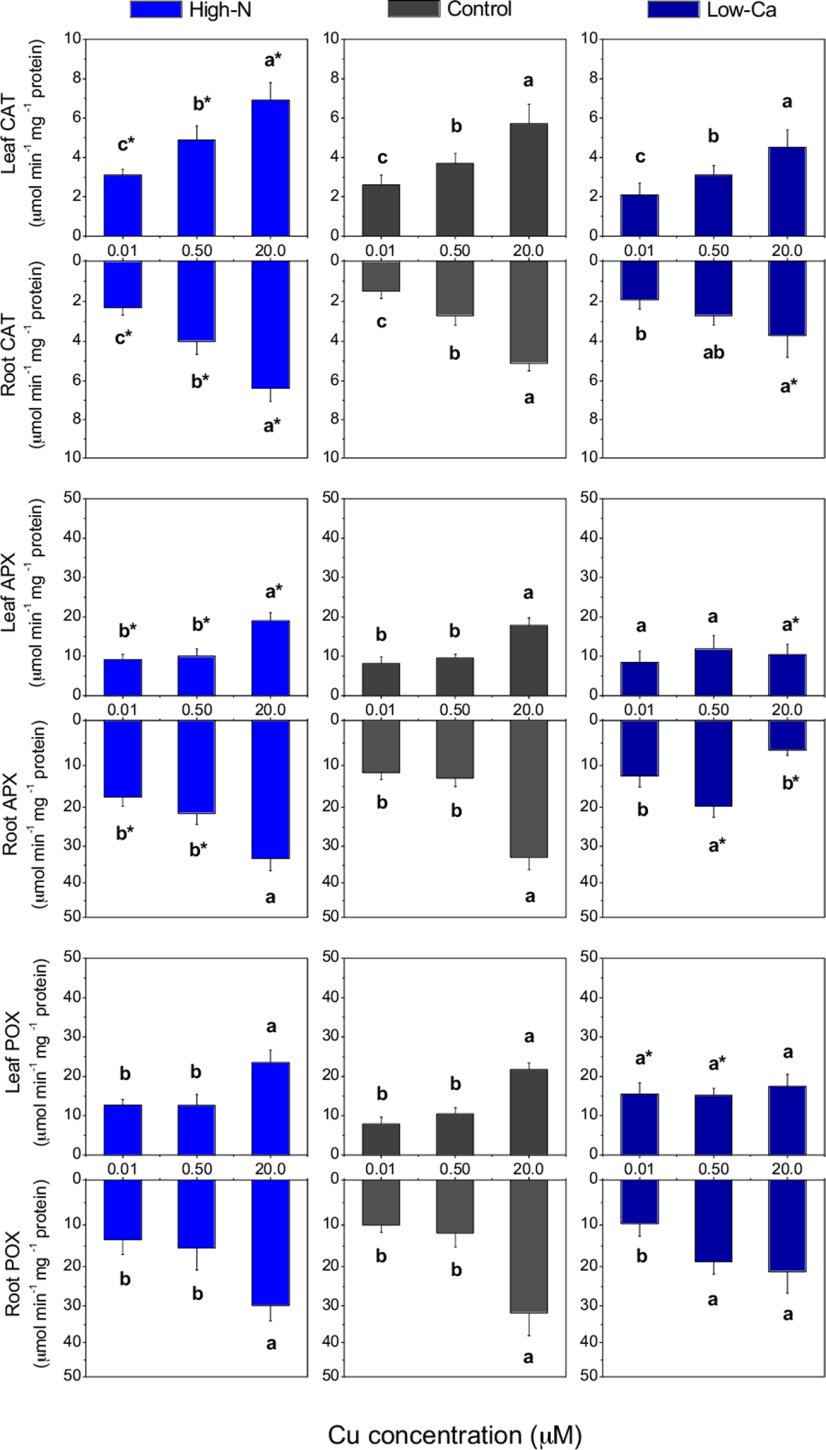
Catalase (CAT), ascorbate peroxidase (APX) and guaiacol peroxidase (POX) activities in Swingle citrumelo seedlings grown in nutrient solution with different levels of nitrogen (N; Experiment 1) or calcium (Ca; Experiment 2), followed by 15 days in various copper (Cu) concentrations. Legend: high-N: 16.4 mM N; control: 9.4 mM N and 5.7 mM Ca; low-Ca: 1.0 mM Ca; Means of Cu levels followed by different lowercase letters are significantly different by Tukey’s test (*p* < 0.05). Copper levels between High-N and Control or between Low-Ca and Control followed by an asterisk (*) are significantly different by Tukey’s test (*p* < 0.05).

The activities of CAT, APX and POX increased in leaves and roots of high-N and control plants grown with 20.0 µM Cu (Fig. 8). These high-N plants also exhibited greater activities of CAT in leaves and roots, APX in roots and POX in leaves than the controls (Fig. 8). In low-Ca plants grown in excess Cu, the activities of CAT, APX and POX decreased, mainly in roots, compared to the control plants (Fig. 8).

## Discussion

Nitrogen and Ca are two key elements for plant nutrition but their potential role in alleviating nutritional stress caused by Cu nutritional disorders in citrus plants is still not well established. There is a need for better fertiliser management practices in citrus production, such as in young and non-bearing trees, in which Cu deficiency frequently occurs, as well as in bearing trees subjected to frequent application of Cu-based pesticides. Thus, in this study we confirmed our hypothesis that adequate management of the N and Ca nutritional status, minimises damages caused by Cu-induced stress in the root medium, mainly by increasing the efficiency of the antioxidant enzyme system, suggesting Cu caused a certain level of oxidative stress.

Although less meaningful responses of treatments were verified on the growth and nutritional status of plants grown under the lowest Cu concentration due to the short-time experiment, the high-N supply decreased the uptake and accumulation of Cu into the roots (Fig. 2). This result confirms the increased Cu demand by plants with high N supply, as previously reported^10^. Under such conditions, visual symptoms of Cu deficiency are exacerbated, which are typically characterised by plant growth with less lignified tissues of new plant parts, enlarged and “S” shaped twigs, and over-developed leaf blades with protruding veins underside^20^.

Copper concentration increased in shoots up to 1.5-fold and in roots up to 10-fold in plants grown with 20.0 µM Cu compared to 0.50 µM Cu (Fig. 2), causing damages to foliar metabolism, such as a decrease in the effective quantum yield of photosystem II (∆F/F_M_′; Fig. 5), chlorophylls and relative water contents^5^. In this study, the decrease in photosynthesis may be explained by an accumulation of ROS in roots and leaves (Fig. 6), further contributing to stomatal closure^21^ and decreasing the carboxylation efficiency (Fig. 4).

Despite showing that the high-N plants with 0.50 µM Cu exhibited no differences in *P*_*N*_/*C*_*i*_ compared to the control, the increased N supply in the NS decreased the *g*_*S*_ and *C*_*i*_ (Fig. 4). Plants with high rather than adequate N supply, also exhibit lower chloroplast CO_2_ concentrations, which limits photosynthesis by limiting ribulose-1, 5-biphosphate carboxylation^22,23^. Furthermore, high-N plants grown in NS with 0.01 or 0.50 µM Cu presented higher AEF values than the controls, probably because of the greater number of electrons destined to the assimilation metabolism of N^22^.

The increased ETR/*P*_*N*_ ratio in the control and low-Ca plants under Cu toxicity, followed by increased *qNP* (Fig. 5), indicates that excessive energy was dissipated in the system. The photochemical (*qP*) or non-photochemical (*qNP*) quenching are responsible for dissipating excessive energy to alleviate the photo-oxidative damages in the photosynthetic apparatus^23^. However, the *qP* and *qNP* were possibly insufficient to avoid damages in the PSII, as verified by the decrease in the ∆F/F_M_′ (Fig. 5), mainly in low-Ca plants, as well by the increased ROS levels (H_2_O_2_ and MDA; Fig. 6).

The specific activities of SOD and APX in roots, CAT in roots and leaves, and POX in leaves of plants exposed to Cu toxicity in both experiments, indicated that the dismutation of superoxide radicals (O_2_^−^), which were generated mainly in the roots, was catalysed by SOD to H_2_O_2_ and the H_2_O_2_ was then eliminated by CAT and APX, also in the roots (Figs 7 and 8). Furthermore, the H_2_O_2_ appeared to be produced and accumulated in leaves, where it was mainly eliminated by CAT and POX (Fig. 8). In this instance, specific stress signals from the roots likely occur, producing and accumulating H_2_O_2_ in roots and leaves^24–26^. Long-distance signalling by H_2_O_2_, by ROS or reactive nitrogen species (RNS), such as nitric oxide, is still not completely understood. The ROS signalling could occur by auto-propagation in waves throughout the plant, in a cell-to-cell communication, and, in this instance, from the root-to-shoot, or by binding to enzymes associated with stress, modifying their activities and coordinating the priming signalling^24,27^.

**Figure 9:**
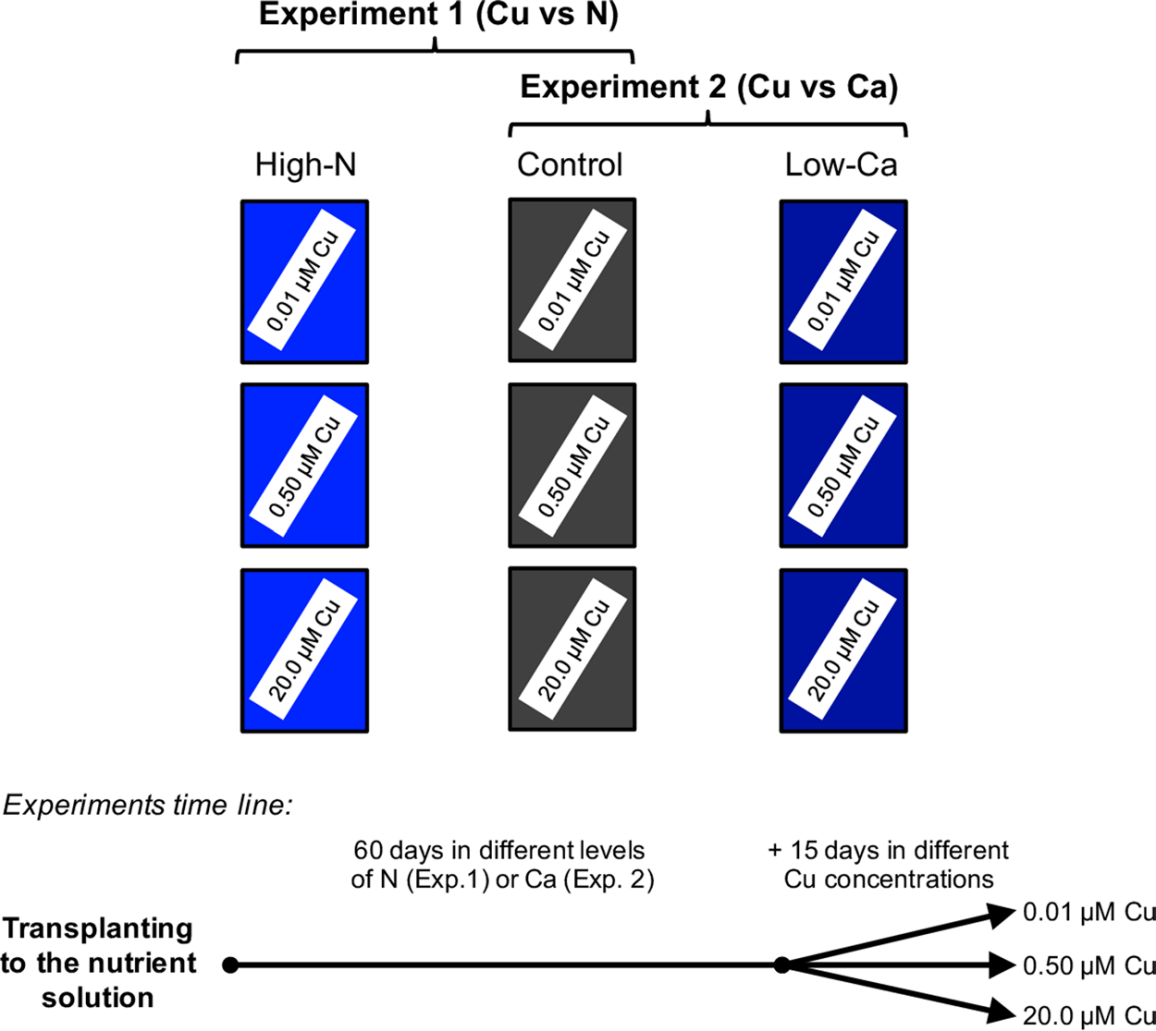
Schematic showing experimental treatments for Swingle citrumelo seedlings grown in nutrient solution with varying nitrogen (N; N-experiment) or calcium (Ca; Ca-experiment) levels for 75 days followed by 15 days in various copper (Cu) concentrations. During the first 60 days of the experiment, plants did not receive Cu supply. Legend: high-N: 16.4 mM N; control (adequate-N and -Ca): 9.4 mM N and 5.7 mM Ca; low-Ca: 1.0 mM Ca.

High-N plants grown in the NS at the highest Cu concentration, accumulated less H_2_O_2_ and MDA and exhibited higher antioxidant enzyme activities than the controls (Fig. 6). Reis *et al*.^14^ reported that in the absence of any abiotic stress, coffee trees did not exhibit enhanced antioxidant enzyme activities with higher N supply. Nevertheless, in this study, high-N citrus plants exhibited lower phytotoxic effect by Cu toxicity because of the greater activities of SOD in roots and CAT in leaves and roots (Figs 8 and 9). Moreover, the decreased phytotoxicity injuries by increased N supply, also probably facilitated the production of organic compounds, such as phytochelatins and metallothioneins, which are cysteine-rich, heavy metal-binding peptides^28,29^.

The NRase activity in leaves was decreased by Cu toxicity (Fig. 3), and increased with increasing N-NO_3_ concentration in shoots of these plants (Supplementary File, Fig. S3), as verified in a previous study of citrus^30^. Thus, we suggest that Cu toxicity affects primarily the N assimilation process. Conversely, excess cadmium (Cd) impairs both NRase activity and nitrate uptake by roots of *Zea mays*^31^. Specific effects of excess Cu on root metabolism have not yet been fully elucidated^32^, indicating the need for further analyses to extend the current knowledge of Cu toxicity effects that disturb N uptake and assimilation.

Calcium is important for a range of structural processes within the cells, such as integrity of membranes and cell walls^17^. Hence, low-Ca plants exhibited higher Cu concentration in the roots compared to control plants both with the highest dose of Cu in the NS (Fig. 2), enhancing damages caused by the metal toxicity, as confirmed by the decrease in *C*_*i*_, ETR and NRase activity (Figs 3 and 4). This macronutrient is also important in another process associated with photosynthesis, namely the organisation of photosynthetic organelles in photosystem II, as well as in the regulation of the opening and closing of stomata via signal transduction pathways^33,34^. The stomatal closure (*g*_*S*_; Fig. 4) was validated by the H_2_O_2_ accumulation in plants grown in NS with high Cu levels (Fig. 6). The Ca channels in the guard cells are activated after increased cytoplasmic Ca^2+^, followed by the transcription of factors and antioxidant enzymes associated with the elimination of ROS^35^. However, in this study, low-Ca plants grown in NS with 20.0 µM Cu exhibited an increased CAT activity, whereas SOD, APX and POX activities were lower than the control plants (Figs 7 and 8). Consequently, a major but indirect participation of CAT in the H_2_O_2_ degradation process, suggests that organelle processes could be more directly affected by a Cu disorder.

Under the low-Ca condition, besides disturbing the antioxidant enzyme system, Cu toxicity, may also have disrupted the non-enzymatic compounds. In citrus seedlings exposed to various Cd levels, the adequate Ca supply promoted glutathione production, essential to the increased phytochelatins levels in root medium, where Cd, also, mostly accumulated^36^.

The rational supply of N and Ca is critical to minimise stress induced by Cu nutritional disorders, mostly associated with toxicity, by limiting the absorption and accumulation of this metal into the plants, as well as by maintaining the integrity of biochemical and physiological processes. The high N supply alleviates ROS-induced damage in plants grown in the NS with high Cu concentrations, due to increased activities of antioxidant enzymes in roots and leaves. Conversely, a low Ca supply decreases the plants metabolism efficiency, resulting in decreased enzymatic and photosynthetic activities.

## Methods

Two experiments were performed in a greenhouse with rootstock Swingle citrumelo [*Citrus paradisi* Macf. x *Poncirus trifoliata* (L.) Raf.] seedlings in NS. Each experiment was constructed in a completely randomised 3 × 2 factorial design, combining three levels of Cu (low: 0.01, medium: 0.50; and high: 20.0 µM, as CuSO_4_.5H_2_O) in the NS with two levels of N (9.4 and 16.4 mM for adequate- and high-N, respectively; referred to as the N-experiment) or two levels of Ca (1.0 and 5.7 mM as low- and adequate-Ca, respectively; referred to as the Ca-experiment), with five replicates. The experiments were performed simultaneously. The plants that received adequate-N and -Ca at the various Cu concentrations were used as the control for both experiments. In the first experiment, plants with high-N level were grown with adequate-Ca in the NS, whereas in the second experiment, plants with low-Ca were grown with adequate-N concentration in the NS.

Six-month-old plants were transplanted into plastic boxes containing 38 L of diluted NS. Initially, plants were adapted to the new growing media at 25% of the final NS concentration in the first week and 50% of the final NS concentration in the second week. For both experiments, the N-NO_3_/N-NH_4_ ratio in the NS was ~8.0. The NS was aerated continuously and constant volumes of solution in the containers were maintained, with deionised water added when required. During the experiment, the NS was maintained at pH ~5.0–5.6 using 1 M KOH or 1 M H_2_SO_4_ and renewed every 15 days.

Plants were maintained in NS containing 0.6 mM P, 3.8 mM potassium (K), 1.8 mM magnesium (Mg), 2.1 mM sulphur (S), 4.63 µM boron (B), 8.95 µM iron (Fe), 0.91 µM manganese (Mn), 0.31 µM zinc (Zn) and 0.05 µM molybdenum (Mo) (modified from Zambrosi *et al*.^5^). Thereafter, plants were separated into two groups to receive the different N or Ca levels, within each experiment as described above, without Cu supply.

After 60 days supplying different levels of N and Ca in the NS, the Cu concentrations were started in the NS (low, adequate and high) for an additional 15-day period, totalling 75 days (Fig. 9). Then, plants were sampled for the evaluation of gas exchange rate, lipid peroxidation (malonaldehyde, MDA) and hydrogen peroxide (H_2_O_2_) contents, and the activities of SOD, CAT, ascorbate peroxidase (APX), POX, and nitrate reductase (NRase).

### Leaf gas exchange and photochemistry activity

The net photosynthetic rate (*P*_*N*_), stomatal conductance to water vapor (*g*_*s*_), internal CO_2_ concentration (*C*_*i*_) and instantaneous transpiration rate (*E*) were determined in the 4^th^–6^th^ expanded leaf from the top and sun-exposed. Evaluations were performed on a clear day between 9:00 and 11:00 h with an open system infrared gas analyser (LI-6400, LI-COR, Lincoln, NE), equipped with an integrated fluorescence chamber head (LI-6400-40, LI-COR), at ambient temperature [0.6215 kPa vapor pressure deficit (VPD)], at 40 Pa CO_2_ partial pressure and under 800 µmol (photon) m^−2^ s^−1^ artificial photosynthetically active photon flux density (*PPFD*) at the leaf level.

Steady-state (F_O_′) and maximum (F_M_′) fluorescence yield were assessed in light-adapted leaf tissues, whereas minimum (F_O_) and maximum (F_M_) fluorescence yield were evaluated in dark-adapted (overnight) leaf tissues. F_M_ and F_M_′ were measured after a light saturation pulse [λ < 710 nm, *PPFD*~10.000 μmol (photon) m^−2^ s^−1^, 0.8 s]. The variable fluorescence yield in both the dark-adapted (F_V_ = F_M_ − F_O_) and light-adapted (F_V_′ = F_M_′ − F_O_′) leaves was calculated. The maximum quantum yield of PSII [F_V_/F_M_ = (F_M_ − F_O_)/F_M_], the effective quantum yield of PSII [∆F/F_M_′ = (F_M_′ − F_S_)/F_M_′], the alternative electron flow [AEF = ∆F/F_M_′/(*P*_*N*_/(*PPFD* × 0.84))], the photochemical quenching coefficient [*qP* = (F_M_′ − F_S_)/(F_M_′ − F_O_′)], and the non-photochemical quenching coefficient [*qNP* = (F_M_ − F_M_′)/F_M_′] were calculated^37^. The apparent electron transport rate [ETR = ∆F/F_M_′ × *PPFD* × 0.84 × 0.5] was calculated according to Genty *et al*.^38^. The ETR/*P*_*N*_ ratio was calculated to estimate the use of electrons in other processes not related to the photosynthetic CO_2_ assimilation rate.

### Activity of NRase

The *in vivo* assay of the NRase activity^39,40^ in leaves was carried out using 100 mg fresh weight of leaves incubated in 100 mM sodium phosphate buffer (pH 7.5), 200 mM KNO_3_ and 1% n-propanol (v/v). A vacuum was applied to the samples for infiltration of the solution into the material and the solutions then incubated at 40 °C for 30 min, in the dark. The NO_2_ formed in the reaction was quantified spectrophotometrically at 540 nm using a 1% sulphanilamide (w/v) solution in 2.4 N HCl and 0.02% *N*-1-naphthyl-ethylene-diamine (w/v). Known amounts of NaNO_2_ were used to construct the calibration curve.

### Contents of H_2_O_2_ and MDA

Measurements of the H_2_O_2_ and MDA content were performed on the same extract, in which 300 mg of leaf or root fresh weight was homogenised in 5 mL of 0.1% (w/v) trichloroacetic acid and centrifuged at 5,590 × *g* at 4 °C for 15 min^41^.

For the determination of H_2_O_2_ content, the supernatant was mixed with 100 mM potassium phosphate buffer (pH 7.0), and 1.0 M potassium iodide (1:1:4) and incubated at 4 °C for 1 h in darkness followed by 25 °C for 20 min. The absorbance of the samples was measured at 390 nm. Known amounts of H_2_O_2_ were used to construct the standard curve.

Lipid peroxidation was determined according to Heath and Packer^42^. A 1 mL solution containing 20% (w/v) trichloroacetic acid and 0.5% (w/v) thiobarbituric acid was added to the supernatant and the mixture was then incubated at 95 °C for 30 min, followed by rapid cooling at 4 °C to stop the reaction. The samples were re-centrifuged at 12,100 × *g* for 5 min and the absorbance of the supernatant was measured at 535 and 600 nm. The amount of MDA [MDA = (A_535_ − A_600_)] was calculated using an extinction coefficient of 155 mM^−1^ cm^−1^.

### Activities of antioxidant enzymes

Leaf or root powder was homogenised in 100 mM potassium phosphate buffer (pH 7.5), with 3 mM dithiothreitol, 1 mM ethylenediaminetetraacetic acid and 4% polyvinylpolypyrrolidone (w/v)^25^. The suspension was centrifuged at 12,100 × *g* at 4 °C for 35 min, and the supernatant was stored at −80 °C for further analysis. The total protein concentration was determined according to Bradford^43^, using bovine serum albumin as a standard.

The SOD activity staining was done^44^ using polyacrylamide (12%) gel electrophoresis under non-denaturing conditions, with 50 µg of protein for leaves and 75 µg for roots samples, loaded in each gel lane. One unit of bovine liver SOD (Sigma, St. Louis, USA) was used as a positive control. The SOD isoforms were distinguished by their sensitivity to inhibition with 2 mM potassium cyanide (KCN) and 5 mM H_2_O_2_^45^. Densitometry analysis of the SOD bands was done according to Tewari *et al*.^46^.

The CAT activity was determined according to Kraus *et al*.^47^, with some modifications^45^. The APX activity was determined by the method of Nakano and Asada^48^ and the POX activity by Kar and Mishra^49^, using pyrogallol as the enzyme substrate^48^, both as described by Hippler *et al*.^30^.

### Plant growth and nutritional status

After the biochemical and physiological sampling (75 days after starting the experiments), plants were harvested destructively for determination of leaf area (LI-3100C, LI-COR, Lincoln, USA), divided into shoots and roots, then washed in distilled water and dried at 58–60 °C till constant dry weight.

Samples were ground to pass through a 200-mesh sieve and nutrient contents (N, Ca and Cu) were determined^50^. The N-NO_3_ and N-NH_4_ levels were determined by steam distillation^51^. From the Cu accumulation in the shoots (CuS) and roots (CuR), the partition ratio of Cu [PR_Cu_ = 100*CuS/(CuS + CuR)] was calculated.

### Statistical analysis

A two-way analysis of variance (ANOVA) was used to evaluate the effect of Cu and N concentrations in the first experiment (N-experiment), as well the effect of Cu and Ca concentrations in the second experiment (Ca-experiment) (PROC ANOVA, SAS version 9.2, SAS Insitute, Cary, NC). If significant interactions between Cu × N (N-experiment) or Cu × Ca (Ca-experiment) were found, mean values were compared using the Tukey test at 5% level

## Electronic supplementary material

Supplementary File
