# Supplementary material for: Oxidative stress induced by Cu nutritional disorders in Citrus depends on nitrogen and calcium availability
Source: Sci Rep. 2018 Jan 26;8:1641. doi: 10.1038/s41598-018-19735-x (PMC5786063; doi:10.1038/s41598-018-19735-x)
Supplement: Supplementary file 1 — Supplementary File [file 41598_2018_19735_MOESM1_ESM.pdf]

Oxidative stress induced by Cu nutritional disorders in *Citrus* depends on nitrogen and calcium availability

Franz Walter Rieger Hippler[1], Rodrigo Marcelli Boaretto[1], Veronica Lorena Dovis[1], José Antônio Quaggio[2], Ricardo Antunes Azevedo[3], Dirceu Mattos-Jr.[1]

[1]Centro de Citricultura Sylvio Moreira, Instituto Agronômico, Rod. Anhanguera, km 158, CP 04, CEP 13490-970, Cordeirópolis, SP, Brazil
[2]Centro de Solos e Recursos Ambientais, Instituto Agronômico, Av. Barão de Itapura, 1481, CP 28, CEP 13020-902, Campinas, SP, Brazil
[3]Departamento de Genética, Escola Superior de Agricultura Luiz de Queiroz, CP 9, Universidade de São Paulo, 13418-900, Piracicaba, SP, Brazil

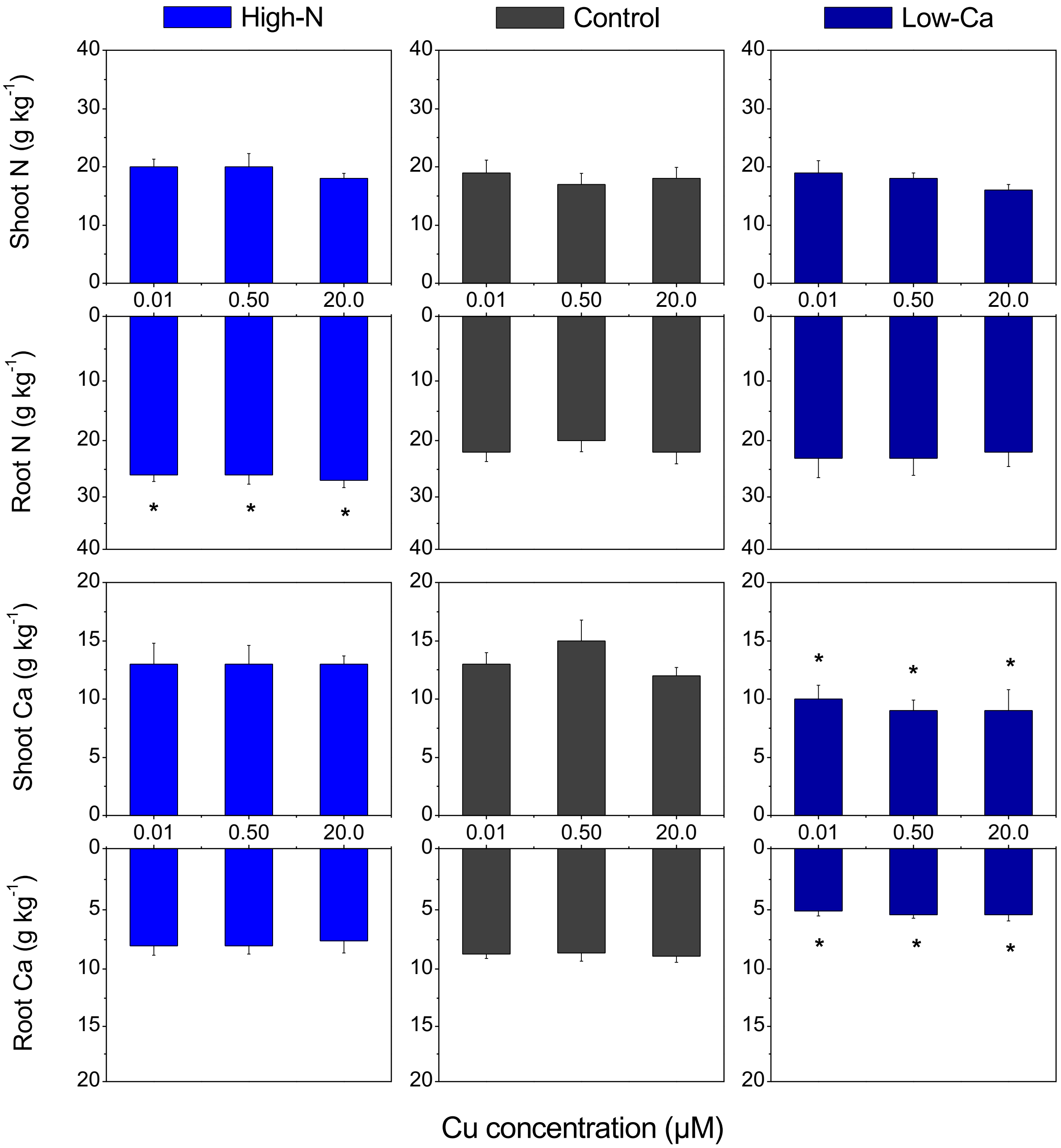

Supplementary Figure S1. Nitrogen (N) and calcium (Ca) concentrations in Swingle citrumelo seedlings grown in nutrient solution with different levels of N (Experiment 1) or Ca (Experiment 2), and followed by 15 days in various copper (Cu) concentrations. Legend: high-N: 16.4 mM N; Control: 9.4 mM N and 5.7 mM Ca; low-Ca: 1.0 mM Ca; Cu levels: means (n=5 or 10) followed by different lowercase letters are significantly different by Tukey's test (p<0.05). N or Ca levels: means (n=5 or 15) followed by an asterisk (*) are significantly different by Tukey's test (p<0.05) when compared to the control.

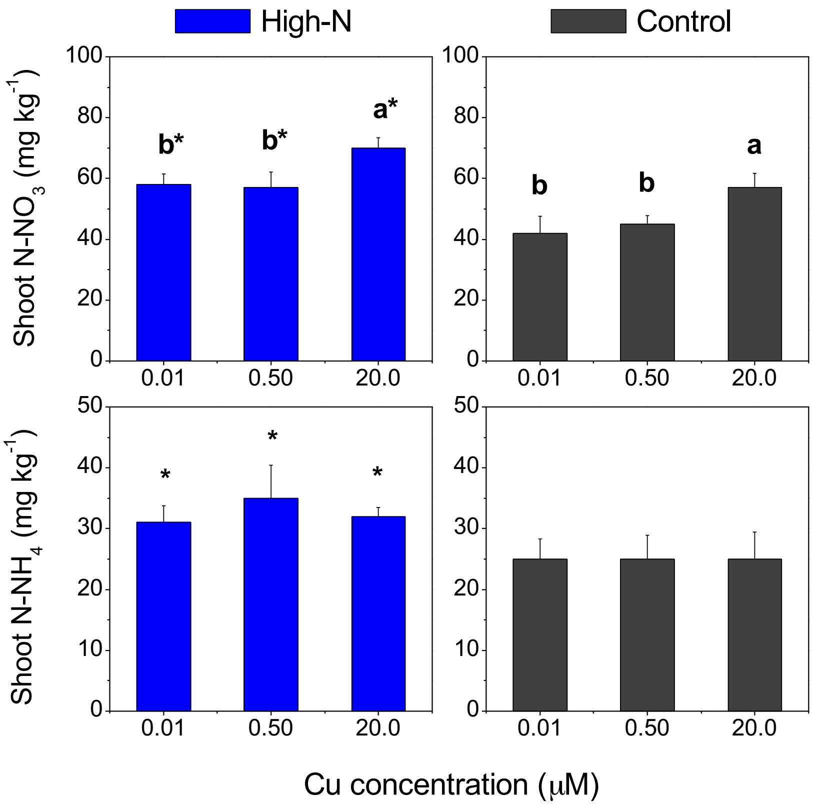

Supplementary Figure S2. Nitrate ($N\text{-}NO_3$) and ammonium ($N\text{-}NH_4$) concentrations in the shoots of Swingle citrumelo seedlings, grown in nutrient solution with different levels of nitrogen (N; Experiment 1), and followed by 15 days in various copper (Cu) concentrations. Legend: high-N: 16.4 mM N; control: 9.4 mM N; Cu levels: means (n=5 or 10) followed by different lowercase letters are significantly different by Tukey's test ($p<0.05$). N levels: means (n=5 or 15) followed by an asterisk (*) are significantly different by Tukey's test ($p<0.05$) when compared to the control.

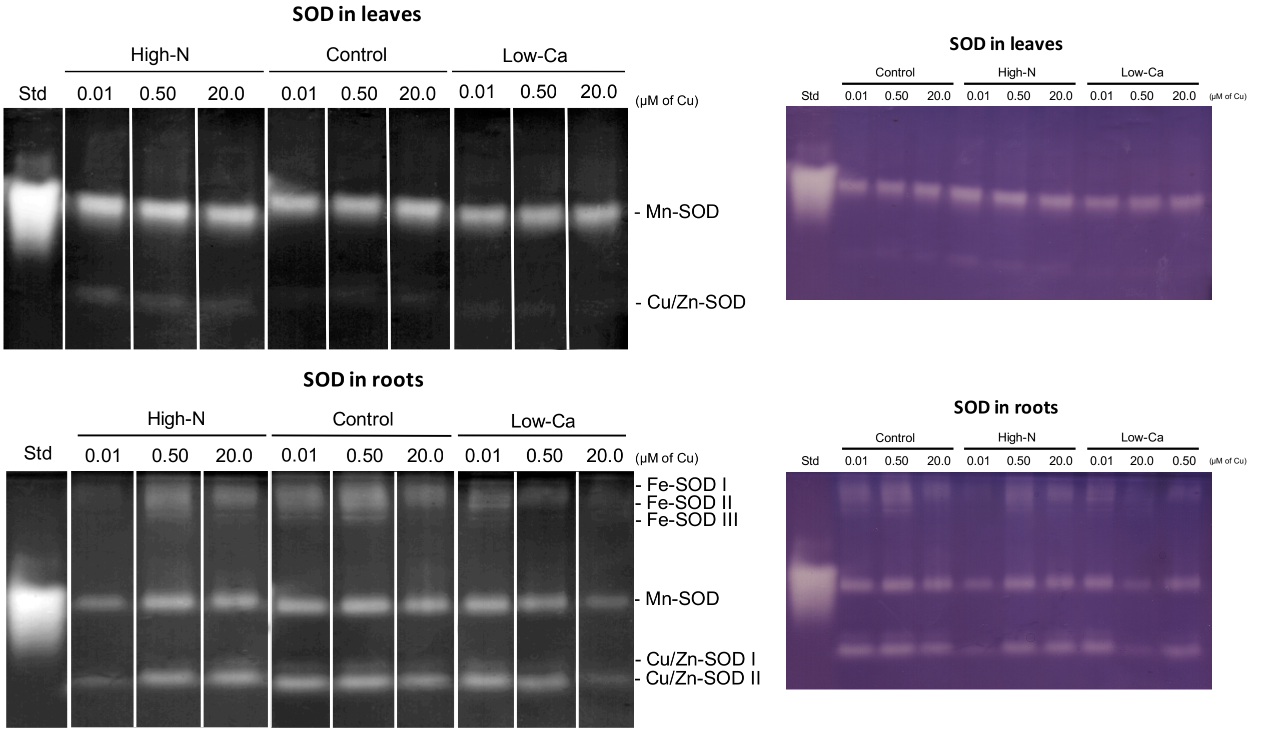

Supplementary Figure S3. Polyacrylamide gel electrophoresis (PAGE 12%) of superoxide dismutase (SOD) activity in leaves and roots of Swingle citrumelo seedlings grown in nutrient solution with different levels of nitrogen (N; Experiment 1) or calcium (Ca; Experiment 2), and followed by 15 days in various copper (Cu) concentrations. Legend: Std – bovine liver SOD standard.

Analysis of variance (ANOVA) for all parameters evaluated in the Experiment 1 - Varying nitrogen and copper levels

Table S1. Anova for dry weight and leaf area data of Swingle citrumelo seedlings grown in nutrient solution with different levels of nitrogen (N; Experiment 1) or calcium (Ca; Experiment 2), followed by 15 days in various copper (Cu) concentrations.

Shoots dry weight

Source	DF	Anova SS	Mean Square	F value	Pr>Fc
Nitrogen	1	0.399053	0.399053	1.299	0.2656
Copper	2	0.033087	0.016543	0.054	0.9477
N*Cu	2	2.543847	1.271923	4.141	0.0285
error	24	7.371560	0.307148		
Corrected total	29	10.347547			

Coeff Var (%) = 17.70
Mean: 3.1313333 Number: 30

Roots dry weight

Source	DF	Anova SS	Mean Square	F value	Pr>Fc
Nitrogen	1	0.074003	0.074003	0.850	0.3658
Copper	2	0.799407	0.399703	4.589	0.0205
N*Cu	2	0.285287	0.142643	1.638	0.2154
error	24	2.090400	0.087100		
Corrected total	29	3.249097			

Coeff Var (%) = 21.39
Mean: 1.3796667 Number: 30

Leaf area

Source	DF	Anova SS	Mean Square	F value	Pr>Fc
Nitrogen	1	107.541333	107.541333	0.292	0.5939
Copper	2	330.764667	165.382333	0.449	0.6435
N*Cu	2	4036.716667	2018.358333	5.480	0.0110
error	24	8840.112000	368.338000		
Corrected total	29	13315.134667			

Coeff Var (%) = 17.35
Mean: 110.5866667 Number: 30

Table S2. Anova for copper (Cu) concentration and partition ratio of Cu (PR_{Cu}) data in Swingle citrumelo seedlings grown in nutrient solution with different levels of nitrogen (N; Experiment 1) or calcium (Ca; Experiment 2), followed by 15 days in various Cu concentrations.

Shoot Cu

Source	DF	Anova SS	Mean Square	F value	Pr>Fc
Nitrogen	1	1.825333	1.825333	2.109	0.1593
Copper	2	42.180667	21.090333	24.372	0.0000
N*Cu	2	1.420667	0.710333	0.821	0.4520
error	24	20.768000	0.865333		
Corrected total	29	66.194667			
Coeff Var (%) =	18.56				
Mean:	5.0133333	Number:	30		

Root Cu

Source	DF	Anova SS	Mean Square	F value	Pr>Fc
Nitrogen	1	736.065333	736.065333	1.920	0.1786
Copper	2	383664.932667	191832.466333	500.401	0.0000
N*Cu	2	698.400667	349.200333	0.911	0.4156
error	24	9200.576000	383.357333		
Corrected total	29	394299.974667			
Coeff Var (%) =	18.50				
Mean:	105.8466667	Number:	30		

Partition ratio of Cu (PR_{Cu})

Source	DF	Anova SS	Mean Square	F value	Pr>Fc
Nitrogen	1	223.041333	223.041333	9.907	0.0044
Copper	2	5208.880667	2604.440333	115.681	0.0000
N*Cu	2	106.360667	53.180333	3.362	0.0458
error	24	540.336000	22.514000		
Corrected total	29	6078.618667			
Coeff Var (%) =	21.46				
Mean:	22.1066667	Number:	30		

Table S3. Anova for nitrate reductase (NRase) activity data in leaves of Swingle citrumelo seedlings, grown in nutrient solution with different levels of nitrogen (N; Experiment 1) or calcium (Ca; Experiment 2), and followed by15 days in various copper (Cu) concentrations.

Nitrate reductase activity (NRase)

Source	DF	Anova SS	Mean Square	F value	Pr>Fc
Nitrogen	1	264807.470083	264807.470083	82.756	0.0000
Copper	2	294714.048447	147357.024223	46.051	0.0000
N*Cu	2	16789.838407	8394.919203	3.624	0.0432
error	24	76797.026400	3199.876100		
Corrected total	29	653108.383337			

Coeff Var (%) = 11.33
Mean: 499.1343333 Number: 30

Table S4. Anova for photosynthetic rate (P_N), stomatal conductance (g_S), internal CO_2 concentration (C_i), apparent electron transport rate (ETR), transpiration (E), instantaneous carboxylation efficiency (P_N/C_i) and ETR/P_N ratio in leaves of Swingle citrumelo seedlings grown in nutrient solution with different levels of nitrogen (N; Experiment 1) or calcium (Ca; Experiment 2), and followed by 15 days in various copper (Cu) concentrations.

Photosynthetic rate (P_N)

Source	DF	Anova SS	Mean Square	F value	Pr>Fc
Nitrogen	1	0.145186	0.145186	0.091	0.7658
Copper	2	61.486762	30.743381	19.226	0.0000
N*Cu	2	10.909283	5.454642	3.411	0.0497
error	24	38.376459	1.599019		
Corrected total	29	110.917691			
Coeff Var (%) =	25.98				
Mean:	4.8681000	Number:	30		

Stomatal conductance (g_S)

Source	DF	Anova SS	Mean Square	F value	Pr>Fc
Nitrogen	1	0.000496	0.000496	2.094	0.1608
Copper	2	0.007135	0.003568	15.060	0.0001
N*Cu	2	0.002873	0.001436	6.063	0.0074
error	24	0.005686	0.000237		
Corrected total	29	0.016190			
Coeff Var (%) =	34.20				
Mean:	0.0450000	Number:	30		

Internal CO_2 concentration (C_i)

Source	DF	Anova SS	Mean Square	F value	Pr>Fc
Nitrogen	1	2078.053350	2078.053350	3.730	0.0653
Copper	2	1299.480759	649.740380	1.166	0.3286
N*Cu	2	6816.210053	3408.105027	6.117	0.0071
error	24	13371.101519	557.129230		
Corrected total	29	23564.845681			
Coeff Var (%) =	12.83				
Mean:	183.9507667	Number:	30		

Instantaneous carboxylation efficiency (P_N/C_i)

Source	DF	Anova SS	Mean Square	F value	Pr>Fc
Nitrogen	1	0.000198	0.000198	5.165	0.0323
Copper	2	0.001678	0.000839	21.928	0.0000
N*Cu	2	0.000212	0.000106	2.976	0.0493
error	24	0.000918	0.000038		
Corrected total	29	0.003007			
Coeff Var (%) =	23.00				
Mean:	0.0269000	Number:	30		

Transpiration (E)

Source	DF	Anova SS	Mean Square	F value	Pr>Fc
Nitrogen	1	0.035639	0.035639	0.749	0.3952
Copper	2	1.487253	0.743626	15.639	0.0000
N*Cu	2	0.436116	0.218058	4.586	0.0206
error	24	1.141214	0.047551		
Corrected total	29	3.100222			
Coeff Var (%) =	30.85				
Mean:	0.7069333	Number:	30		

Table S5. Anova for apparent electron transport rate (ETR), ratio between ETR and CO_2 assimilation (ETR/P_N), alternative electron flow (AEF), the effective quantum yield of photosystem II ($\Delta F/F_M$'), and the photochemical (*qP*) and non-photochemical quenching (*qNP*) data in leaves of Swingle citrumelo seedlings grown in nutrient solution with different levels of nitrogen (N; Experiment 1) or calcium (Ca; Experiment 2), and followed by 15 days in various copper (Cu) concentrations.

Apparent electron transport rate (ETR)

Source	DF	Anova SS	Mean Square	F value	Pr>Fc
Nitrogen	1	30.122124	30.122124	0.881	0.3573
Copper	2	1663.793191	831.896595	24.331	0.0000
N*Cu	2	443.214058	221.607029	6.482	0.0056
error	24	820.573092	34.190546		
Corrected total	29	2957.702465			
Coeff Var (%) =	10.13				
Mean:	57.7360333	Number:	30		

Ratio between ETR and CO_2 assimilation (ETR/P_N)

Source	DF	Anova SS	Mean Square	F value	Pr>Fc
Nitrogen	1	10.917920	10.917920	0.457	0.5057
Copper	2	179.757862	89.878931	3.758	0.0380
N*Cu	2	44.473844	22.236922	0.930	0.4084
error	24	573.958663	23.914944		
Corrected total	29	809.108290			
Coeff Var (%) =	36.74				
Mean:	13.3120000	Number:	30		

Alternative electron flow (AEF)

Source	DF	Anova SS	Mean Square	F value	Pr>Fc
Nitrogen	1	609.175153	609.175153	44.634	0.0000
Copper	2	2426.950229	1213.475114	88.911	0.0000
N*Cu	2	263.412552	131.706276	9.650	0.0008
error	24	327.558437	13.648268		
Corrected total	29	3627.096371			
Coeff Var (%) =	10.60				
Mean:	34.8441333	Number:	30		

The effective quantum yield of photosystem II ($\Delta F/F_M'$)

Source	DF	Anova SS	Mean Square	F value	Pr>Fc
Nitrogen	1	0.000320	0.000320	1.077	0.3097
Copper	2	0.014015	0.007007	23.577	0.0000
N*Cu	2	0.004021	0.002011	6.765	0.0047
error	24	0.007133	0.000297		
Corrected total	29	0.025489			
Coeff Var (%) =	10.17				
Mean:	0.1694667	Number:	30		

Photochemical quenching (qP)

Source	DF	Anova SS	Mean Square	F value	Pr>Fc
Nitrogen	1	0.008069	0.008069	3.923	0.0592
Copper	2	0.007446	0.003723	1.810	0.1853
N*Cu	2	0.012152	0.006076	2.954	0.0713
error	24	0.049366	0.002057		
Corrected total	29	0.077033			
Coeff Var (%) =	11.50				
Mean:	0.3943333	Number:	30		

Non-photochemical quenching (qNP)

Source	DF	Anova SS	Mean Square	F value	Pr>Fc
Nitrogen	1	0.004465	0.004465	0.034	0.8550
Copper	2	3.665203	1.832602	14.013	0.0001
N*Cu	2	1.440858	0.720429	5.509	0.0107
error	24	3.138609	0.130775		
Corrected total	29	8.249135			
Coeff Var (%) =	19.10				
Mean:	1.8934667	Number:	30		

Table S6. Anova for hydrogen peroxide (H_2O_2) concentration and lipid peroxidation (malonaldehyde, MDA) data in Swingle citrumelo seedlings grown in nutrient solution with different levels of nitrogen (N; Experiment 1) or calcium (Ca; Experiment 2), and followed by 15 days in various copper (Cu) concentrations.

Leaf H_2O_2

Source	DF	Anova SS	Mean Square	F value	Pr>Fc
Nitrogen	1	0.563070	0.563070	9.738	0.0047
Copper	2	7.357820	3.678910	63.627	0.0000
N*Cu	2	0.434660	0.217330	3.759	0.0380
error	24	1.387680	0.057820		
Corrected total	29	9.743230			
Coeff Var (%) =	15.19				
Mean:	1.5830000	Number:	30		

Root H_2O_2

Source	DF	Anova SS	Mean Square	F value	Pr>Fc
Nitrogen	1	0.007053	0.007053	12.023	0.0020
Copper	2	0.060807	0.030403	51.824	0.0000
N*Cu	2	0.004407	0.002203	3.756	0.0381
error	24	0.014080	0.000587		
Corrected total	29	0.086347			
Coeff Var (%) =	18.82				
Mean:	0.1286667	Number:	30		

Leaf MDA

Source	DF	Anova SS	Mean Square	F value	Pr>Fc
Nitrogen	1	0.000853	0.000853	0.102	0.7527
Copper	2	1.938007	0.969003	115.358	0.0000
N*Cu	2	0.122127	0.061063	7.269	0.0034
error	24	0.201600	0.008400		
Corrected total	29	2.262587			
Coeff Var (%) =	11.74				
Mean:	0.7806667	Number:	30		

Root MDA

Source	DF	Anova SS	Mean Square	F value	Pr>Fc
Nitrogen	1	0.002803	0.002803	0.787	0.3838
Copper	2	0.318647	0.159323	44.733	0.0000
N*Cu	2	0.024407	0.012203	3.426	0.0491
error	24	0.085480	0.003562		
Corrected total	29	0.431337			
Coeff Var (%) =	18.21				
Mean:	0.3276667	Number:	30		

Table S8. Anova for catalase (CAT), ascorbate peroxidase (APX) and guaiacol peroxidase (POX) activities data in Swingle citrumelo seedlings grown in nutrient solution with different levels of nitrogen (N; Experiment 1) or calcium (Ca; Experiment 2), followed by 15 days in various copper (Cu) concentrations.

Leaf CAT

Source	DF	Anova SS	Mean Square	F value	Pr>Fc
Nitrogen	1	9.229653	9.229653	25.968	0.0000
Copper	2	52.729620	26.364810	74.178	0.0000
N*Cu	2	0.117167	0.058583	0.165	0.8490
error	24	8.530280	0.355428		
Corrected total	29	70.606720			
Coeff Var (%) =	13.06				
Mean:	4.5640000	Number:	30		

Root CAT

Source	DF	Anova SS	Mean Square	F value	Pr>Fc
Nitrogen	1	9.218563	9.218563	47.341	0.0000
Copper	2	73.849447	36.924723	189.625	0.0000
N*Cu	2	0.446607	0.223303	1.147	0.3345
error	24	4.673400	0.194725		
Corrected total	29	88.188017			
Coeff Var (%) =	12.05				
Mean:	3.6616667	Number:	30		

Leaf APX

Source	DF	Anova SS	Mean Square	F value	Pr>Fc
Nitrogen	1	6.265470	6.265470	3.172	0.0476
Copper	2	576.482247	288.241123	136.723	0.0000
N*Cu	2	0.708500	0.354250	0.168	0.8463
erro	24	50.597200	2.108217		
Corrected total	29	634.053417			
Coeff Var (%) =	11.84				
Mean:	12.2616667	Number:	30		

Root APX

Source	DF	Anova SS	Mean Square	F value	Pr>Fc
Nitrogen	1	178.266563	178.266563	8.422	0.0078
Copper	2	1988.618660	994.309330	46.973	0.0000
N*Cu	2	88.257647	44.128823	2.085	0.1463
error	24	508.021280	21.167553		
Corrected total	29	2763.164150			
Coeff Var (%) =	21.25				
Mean:	21.6550000	Number:	30		

Leaf POX

Source	DF	Anova SS	Mean Square	F value	Pr>Fc
Nitrogen	1	62.507881	62.507881	17.420	0.0003
Copper	2	911.286263	455.643132	126.978	0.0000
N*Cu	2	13.636782	6.818391	1.900	0.1714
error	24	86.120904	3.588371		
Corrected total	29	1073.551830			

Coeff Var (%) = 12.83
Mean: 14.7609333 Number: 30

Root POX

Source	DF	Anova SS	Mean Square	F value	Pr>Fc
Nitrogen	1	18.767266	18.767266	1.352	0.2564
Copper	2	2217.601847	1108.800924	79.851	0.0000
N*Cu	2	50.180477	25.090239	1.807	0.1858
error	24	333.259691	13.885820		
Corrected total	29	2619.809281			

Coeff Var (%) = 19.98
Mean: 18.6504667 Number: 30

Table S9. Anova for nitrogen (N) and calcium (Ca) concentrations data in Swingle citrumelo seedlings grown in nutrient solution with different levels of N (Experiment 1) or Ca (Experiment 2), and followed by 15 days in various copper (Cu) concentrations.

Shoot N

Source	DF	Anova SS	Mean Square	F value	Pr>Fc
Nitrogen	1	21.168000	21.168000	6.645	0.0165
Copper	2	6.264667	3.132333	0.983	0.3886
N*Cu	2	6.498000	3.249000	1.020	0.3757
error	24	76.448000	3.185333		
Corrected total	29	110.378667			
Coeff Var (%) =	9.56				
Mean:	18.6733333	Number:	30		

Root N

Source	DF	Anova SS	Mean Square	F value	Pr>Fc
Nitrogen	1	33.708000	33.708000	4.315	0.0486
Copper	2	11.330667	5.665333	0.725	0.4945
N*Cu	2	5.928000	2.964000	0.379	0.6883
error	24	187.472000	7.811333		
Corrected total	29	238.438667			
Coeff Var (%) =	11.41				
Mean:	24.4933333	Number:	30		

Shoot Ca

Source	DF	Anova SS	Mean Square	F value	Pr>Fc
Nitrogen	1	5.043000	5.043000	2.438	0.1315
Copper	2	7.900667	3.950333	1.910	0.1700
N*Cu	2	7.358000	3.679000	1.778	0.1904
error	24	49.648000	2.068667		
Corrected total	29	69.949667			
Coeff Var (%) =	10.95				
Mean:	13.1366667	Number:	30		

Root Ca

Source	DF	Anova SS	Mean Square	F value	Pr>Fc
Nitrogen	1	6.165333	6.165333	2.999	0.0520
Copper	2	0.040667	0.020333	0.040	0.9613
N*Cu	2	0.880667	0.440333	0.857	0.4370
error	24	12.332000	0.513833		
Corrected total	29	19.418667			
Coeff Var (%) =	8.64				
Mean:	8.2933333	Number:	30		

Analysis of variance (ANOVA) for all parameters evaluated in the Experiment 2 - Varying calcium and copper levels

Table S10. Anova for dry weight and leaf area data of Swingle citrumelo seedlings grown in nutrient solution with different levels of nitrogen (N; Experiment 1) or calcium (Ca; Experiment 2), followed by 15 days in various copper (Cu) concentrations.

Shoots dry weight

Source	DF	Anova SS	Mean Square	F value	Pr>Fc
Calcium	1	0.003000	0.003000	0.014	0.9066
Copper	2	1.280220	0.640110	3.904	0.0321
Ca*Cu	2	0.321860	0.160930	0.755	0.4809
error	24	5.115840	0.213160		
Corrected total	29	6.720920			
Coeff Var (%) =	15.36				
Mean:	3.0060000	Number:	30		

Roots dry weight

Source	DF	Anova SS	Mean Square	F value	Pr>Fc
Calcium	1	0.012403	0.012403	0.288	0.5963
Copper	2	0.455887	0.227943	5.296	0.0124
Ca*Cu	2	0.474327	0.237163	5.511	0.0107
error	24	1.032880	0.043037		
Corrected total	29	1.975497			
Coeff Var (%) =	15.84				
Mean:	1.3096667	Number:	30		

Leaf area

Source	DF	Anova SS	Mean Square	F value	Pr>Fc
Calcium	1	295.788000	295.788000	0.826	0.3726
Copper	2	1996.248667	998.124333	3.586	0.0316
Ca*Cu	2	802.982000	401.491000	3.121	0.0425
error	24	8598.136000	358.255667		
Corrected total	29	11693.154667			
Coeff Var (%) =	17.93				
Mean:	105.5533333	Number:	30		

Table S11. Anova for copper (Cu) concentration and partition ratio of Cu (PR_{Cu}) data in Swingle citrumelo seedlings grown in nutrient solution with different levels of nitrogen (N; Experiment 1) or calcium (Ca; Experiment 2), followed by 15 days in various Cu concentrations.

Shoot Cu

Source	DF	Anova SS	Mean Square	F value	Pr>Fc
Calcium	1	1.776333	1.776333	2.164	0.1543
Copper	2	32.500667	16.250333	19.793	0.0000
Ca*Cu	2	0.240667	0.120333	0.147	0.8644
error	24	19.704000	0.821000		
Corrected total	29	54.221667			
Coeff Var (%) =	18.06				
Mean:	5.0166667	Number:	30		

Root Cu

Source	DF	Anova SS	Mean Square	F value	Pr>Fc
Calcium	1	2150.533333	2150.533333	4.362	0.0475
Copper	2	494860.850667	247430.425333	501.834	0.0000
Ca*Cu	2	10792.674667	5396.337333	10.945	0.0004
error	24	11833.248000	493.052000		
Corrected total	29	519637.306667			
Coeff Var (%) =	18.62				
Mean:	119.2666667	Number:	30		

Partition ratio of Cu (PR_{Cu})

Source	DF	Anova SS	Mean Square	F value	Pr>Fc
Calcium	1	37.856333	37.856333	2.367	0.1370
Copper	2	4342.244667	2171.122333	135.774	0.0000
Ca*Cu	2	106.972667	53.486333	3.345	0.0493
error	24	383.776000	15.990667		
Corrected total	29	4870.849667			
Coeff Var (%) =	19.50				
Mean:	20.5033333	Number:	30		

Table S12. Anova for nitrate reductase (NRase) activity data in leaves of Swingle citrumelo seedlings, grown in nutrient solution with different levels of nitrogen (N; Experiment 1) or calcium (Ca; Experiment 2), and followed by15 days in various copper (Cu) concentrations.

Nitrate reductase activity (NRase)

Source	DF	Anova SS	Mean Square	F value	Pr>Fc
Calcium	1	113929.218750	113929.218750	52.502	0.0000
Copper	2	128468.069047	64234.034523	29.601	0.0000
Ca*Cu	2	19520.488860	9760.244430	4.498	0.0219
error	24	52079.755880	2169.989828		
Corrected total	29	313997.532537			
Coeff Var (%) =	13.56				
Mean:	343.5576667	Number:	30		

Table S13. Anova for photosynthetic rate (P_N), stomatal conductance (g_S), internal CO_2 concentration (C_i), apparent electron transport rate (ETR), transpiration (E), instantaneous carboxylation efficiency (P_N/C_i) and ETR/P_N ratio data in leaves of Swingle citrumelo seedlings grown in nutrient solution with different levels of nitrogen (N; Experiment 1) or calcium (Ca; Experiment 2), and followed by 15 days in various copper (Cu) concentrations.

Photosynthetic rate (P_N)

Source	DF	Anova SS	Mean Square	F value	Pr>Fc
Calcium	1	0.244803	0.244803	0.188	0.6686
Copper	2	143.044247	71.522123	54.867	0.0000
Ca*Cu	2	2.936327	1.468163	1.126	0.3408
error	24	31.285160	1.303548		
Corrected total	29	177.510537			
Coeff Var (%) =	24.25				
Mean:	4.7076667	Number:	30		

Stomatal conductance (g_S)

Source	DF	Anova SS	Mean Square	F value	Pr>Fc
Calcium	1	0.000626	0.000626	2.883	0.1025
Copper	2	0.017379	0.008689	40.040	0.0000
Ca*Cu	2	0.000043	0.000021	0.099	0.9063
error	24	0.005208	0.000217		
Corrected total	29	0.023256			
Coeff Var (%) =	33.10				
Mean:	0.0445000	Number:	30		

Internal CO_2 concentration (C_i)

Source	DF	Anova SS	Mean Square	F value	Pr>Fc
Calcium	1	121.203000	121.203000	0.140	0.7120
Copper	2	11081.192667	5540.596333	6.381	0.0060
Ca*Cu	2	3935.774000	1967.887000	3.266	0.0454
error	24	20839.600000	868.316667		
Corrected total	29	35977.769667			
Coeff Var (%) =	15.49				
Mean:	119.2666667	Number:	30		

Instantaneous carboxylation efficiency (P_N/C_i)

Source	DF	Anova SS	Mean Square	F value	Pr>Fc
Calcium	1	0.000004	0.000004	0.095	0.7609
Copper	2	0.002307	0.001153	27.094	0.0000
Ca*Cu	2	0.000154	0.000077	1.810	0.1853
error	24	0.001022	0.000043		
Corrected total	29	0.003486			
Coeff Var (%) =	26.41				
Mean:	0.0247000	Number:	30		

Transpiration (E)

Source	DF	Anova SS	Mean Square	F value	Pr>Fc
Calcium	1	0.010830	0.010830	0.219	0.6443
Copper	2	4.099220	2.049610	41.385	0.0000
Ca*Cu	2	0.036780	0.018390	0.371	0.6937
error	24	1.188600	0.049525		
Corrected total	29	5.335430			
Coeff Var (%) =	30.78				
Mean:	0.7230000	Number:	30		

Table S14. Anova for apparent electron transport rate (ETR), ratio between ETR and CO_2 assimilation (ETR/P_N), alternative electron flow (AEF), the effective quantum yield of photosystem II ($\Delta F/F_M$'), and the photochemical (*qP*) and non-photochemical quenching (*qNP*) data in leaves of Swingle citrumelo seedlings grown in nutrient solution with different levels of nitrogen (N; Experiment 1) or calcium (Ca; Experiment 2), and followed by 15 days in various copper (Cu) concentrations.

Apparent electron transport rate (ETR)

Source	DF	Anova SS	Mean Square	F value	Pr>Fc
Calcium	1	176.758413	176.758413	1.679	0.2074
Copper	2	4859.193087	2429.596543	23.072	0.0000
Ca*Cu	2	1859.727847	929.863923	8.830	0.0013
error	24	2527.341320	105.305888		
Corrected total	29	9423.020667			
Coeff Var (%) =	18.90				
Mean:	54.3066667	Number:	30		

Ratio between ETR and CO_2 assimilation (ETR/P_N)

Source	DF	Anova SS	Mean Square	F value	Pr>Fc
Calcium	1	16.118670	16.118670	1.260	0.2727
Copper	2	304.358827	152.179413	11.897	0.0003
Ca*Cu	2	53.431440	26.715720	2.089	0.1458
error	24	306.999000	12.791625		
Corrected total	29	680.907937			
Coeff Var (%) =	27.13				
Mean:	13.1823333	Number:	30		

Alternative electron flow (AEF)

Source	DF	Anova SS	Mean Square	F value	Pr>Fc
Calcium	1	634.800000	634.800000	52.621	0.0000
Copper	2	4759.754667	2379.877333	197.276	0.0000
Ca*Cu	2	13.272000	6.636000	0.550	0.5840
error	24	289.528000	12.063667		
Corrected total	29	5697.354667			
Coeff Var (%) =	9.94				
Mean:	34.9466667	Number:	30		

The effective quantum yield of photosystem II ($\Delta F/F_M$')

Source	DF	Anova SS	Mean Square	F value	Pr>Fc
Calcium	1	0.001360	0.001360	1.497	0.2331
Copper	2	0.042549	0.021275	23.411	0.0000
Ca*Cu	2	0.015872	0.007936	8.733	0.0014
error	24	0.021810	0.000909		
Corrected total	29	0.081591			
Coeff Var (%) =	18.90				
Mean:	0.1594667	Number:	30		

Photochemical quenching (qP)

Source	DF	Anova SS	Mean Square	F value	Pr>Fc
Calcium	1	0.013063	0.013063	2.107	0.1596
Copper	2	0.072657	0.036329	5.859	0.0085
Ca*Cu	2	0.104909	0.052454	8.460	0.0017
error	24	0.148799	0.006200		
Corrected total	29	0.339428			

Coeff Var (%) = 22.05
Mean: 0.3570667 Number: 30

Non-photochemical quenching (qNP)

Source	DF	Anova SS	Mean Square	F value	Pr>Fc
Calcium	1	0.504403	0.504403	3.822	0.0623
Copper	2	4.689380	2.344690	17.769	0.0000
Ca*Cu	2	1.102687	0.551343	4.178	0.0277
error	24	3.166960	0.131957		
Corrected total	29	9.463430			

Coeff Var (%) = 20.72
Mean: 1.7530000 Number: 30

Table S15. Anova for hydrogen peroxide (H_2O_2) concentration and lipid peroxidation (malonaldehyde, MDA) data in Swingle citrumelo seedlings grown in nutrient solution with different levels of nitrogen (N; Experiment 1) or calcium (Ca; Experiment 2), and followed by 15 days in various copper (Cu) concentrations.

Leaf H_2O_2

Source	DF	Anova SS	Mean Square	F value	Pr>Fc
Calcium	1	1.075413	1.075413	63.577	0.0000
Copper	2	9.479787	4.739893	280.218	0.0000
Ca*Cu	2	0.096027	0.048013	3.039	0.0483
error	24	0.405960	0.016915		
Corrected total	29	11.057187			
Coeff Var (%) =	6.81				
Mean:	1.9093333	Number:	30		

Root H_2O_2

Source	DF	Anova SS	Mean Square	F value	Pr>Fc
Calcium	1	0.008333	0.008333	21.930	0.0001
Copper	2	0.120987	0.060493	159.193	0.0000
Ca*Cu	2	0.001947	0.000973	3.061	0.0481
error	24	0.009120	0.000380		
Corrected total	29	0.140387			
Coeff Var (%) =	12.13				
Mean:	0.1606667	Number:	30		

Leaf MDA

Source	DF	Anova SS	Mean Square	F value	Pr>Fc
Calcium	1	0.538680	0.538680	54.202	0.0000
Copper	2	2.379620	1.189810	119.719	0.0000
Ca*Cu	2	0.038780	0.019390	1.951	0.1640
error	24	0.238520	0.009938		
Corrected total	29	3.195600			
Coeff Var (%) =	10.84				
Mean:	0.9200000	Number:	30		

Root MDA

Source	DF	Anova SS	Mean Square	F value	Pr>Fc
Calcium	1	0.027000	0.027000	9.441	0.0052
Copper	2	0.304287	0.152143	53.197	0.0000
Ca*Cu	2	0.014660	0.007330	3.263	0.0420
error	24	0.068640	0.002860		
Corrected total	29	0.414587			
Coeff Var (%) =	14.56				
Mean:	0.3673333	Number:	30		

Table S16. Anova for catalase (CAT), ascorbate peroxidase (APX) and guaiacol peroxidase (POX) activities data in Swingle citrumelo seedlings grown in nutrient solution with different levels of nitrogen (N; Experiment 1) or calcium (Ca; Experiment 2), followed by 15 days in various copper (Cu) concentrations.

Leaf CAT

Source	DF	Anova SS	Mean Square	F value	Pr>Fc
Calcium	1	4.531853	4.531853	13.126	0.0014
Copper	2	37.762667	18.881333	54.690	0.0000
Ca*Cu	2	0.932587	0.466293	1.351	0.2781
error	24	8.285880	0.345245		
Corrected total	29	51.512987			
Coeff Var (%) =	16.23				
Mean:	3.6206667	Number:	30		

Root CAT

Source	DF	Anova SS	Mean Square	F value	Pr>Fc
Calcium	1	1.761763	1.761763	6.527	0.0174
Copper	2	31.013460	15.506730	57.446	0.0000
Ca*Cu	2	6.773087	3.386543	12.546	0.0002
error	24	6.478440	0.269935		
Corrected total	29	46.026750			
Coeff Var (%) =	18.13				
Mean:	2.8650000	Number:	30		

Leaf APX

Source	DF	Anova SS	Mean Square	F value	Pr>Fc
Calcium	1	18.738803	18.738803	4.307	0.0488
Copper	2	172.480047	86.240023	19.820	0.0000
Ca*Cu	2	136.391607	68.195803	15.673	0.0000
erro	24	104.427480	4.351145		
Corrected total	29	432.037937			
Coeff Var (%) =	19.94				
Mean:	11.0143333	Number:	30		

Root APX

Source	DF	Anova SS	Mean Square	F value	Pr>Fc
Calcium	1	296.479203	296.479203	18.022	0.0003
Copper	2	288.698807	144.349403	8.775	0.0014
Ca*Cu	2	1565.071047	782.535523	47.568	0.0000
error	24	394.819240	16.450802		
Corrected total	29	2545.068297			
Coeff Var (%) =	25.23				
Mean:	16.0736667	Number:	30		

Leaf POX

Source	DF	Anova SS	Mean Square	F value	Pr>Fc
Calcium	1	25.208333	25.208333	3.561	0.0713
Copper	2	274.272000	137.136000	19.372	0.0000
Ca*Cu	2	264.962667	132.481333	18.714	0.0000
error	24	169.900000	7.079167		
Corrected total	29	734.343000			
Coeff Var (%) =	18.70				
Mean:	14.2300000	Number:	30		

Root POX

Source	DF	Anova SS	Mean Square	F value	Pr>Fc
Calcium	1	50.440333	50.440333	0.998	0.3277
Copper	2	520.088000	260.044000	5.146	0.0138
Ca*Cu	2	110.378667	55.189333	1.092	0.3516
error	24	1212.880000	50.536667		
Corrected total	29	1893.787000			
Coeff Var (%) =	41.55				
Mean:	17.1100000	Number:	30		

Table S17. Anova for nitrogen (N) and calcium (Ca) concentrations data in Swingle citrumelo seedlings grown in nutrient solution with different levels of N (Experiment 1) or Ca (Experiment 2), and followed by 15 days in various copper (Cu) concentrations.

Shoot N

Source	DF	Anova SS	Mean Square	F value	Pr>Fc
Calcium	1	1.008333	1.008333	0.355	0.5571
Copper	2	12.128000	6.064000	2.132	0.1405
Ca*Cu	2	5.266667	2.633333	0.926	0.4098
error	24	68.252000	2.843833		
Corrected total	29	86.655000			
Coeff Var (%) =	9.55				
Mean:	17.6500000	Number:	30		

Root N

Source	DF	Anova SS	Mean Square	F value	Pr>Fc
Calcium	1	20.667000	20.667000	2.155	0.1551
Copper	2	15.028667	7.514333	0.783	0.4682
Ca*Cu	2	12.978000	6.489000	0.676	0.5178
error	24	230.216000	9.592333		
Corrected total	29	278.889667			
Coeff Var (%) =	12.76				
Mean:	24.2633333	Number:	30		

Shoot Ca

Source	DF	Anova SS	Mean Square	F value	Pr>Fc
Calcium	1	77.763000	77.763000	39.284	0.0000
Copper	2	4.932667	2.466333	1.246	0.3056
Ca*Cu	2	12.566000	6.283000	3.174	0.0598
error	24	47.508000	1.979500		
Corrected total	29	142.769667			
Coeff Var (%) =	11.79				
Mean:	11.9366667	Number:	30		

Root Ca

Source	DF	Anova SS	Mean Square	F value	Pr>Fc
Calcium	1	70.533333	70.533333	297.609	0.0000
Copper	2	0.044667	0.022333	0.094	0.9104
Ca*Cu	2	0.588667	0.294333	1.242	0.3067
error	24	5.688000	0.237000		
Corrected total	29	76.854667			
Coeff Var (%) =	6.75				
Mean:	7.2133333	Number:	30		
